# Cognitive Abilities and Educational Attainment as Antecedents of Mental Disorders: A Total Population Study of Males

**DOI:** 10.1177/09567976251347221

**Published:** 2025-06-26

**Authors:** Magnus Nordmo, Hans Fredrik Sunde, Thomas H. Kleppestø, Morten Nordmo, Avshalom Caspi, Terrie E. Moffitt, Fartein Ask Torvik

**Affiliations:** 1 Department of Educational Science, University of South-Eastern Norway; 2 Centre for Fertility and Health, Norwegian Institute of Public Health; 3 Department of Psychology, Norwegian University of Science and Technology; 4 Norwegian Business School, Department of Leadership and Organizational Behavior; 5 Department of Psychology and Neuroscience, Duke University; 6 Promenta Research Center, Department of Psychology, University of Oslo; 7 Social Genetic and Developmental Psychiatry Centre, Institute of Psychology, Psychiatry, and Neuroscience, King’s College London

**Keywords:** cognitive abilities, educational attainment, mental disorders, intelligence

## Abstract

The positive relation between mental health and educational attainment is well established, yet the extent to which cognitive abilities influence this gradient or independently predict mental health outcomes remains unclear. In this study, we investigated the association between adolescent cognitive abilities, educational attainment, and adult mental health. Cognitive ability was ascertained in Norwegian military conscript test data (*N* = 272,351; mean age 17.8 years; males only), whereas mental disorders were ascertained using the Norwegian register of primary care diagnoses received between the age of 36–40. Higher cognitive abilities were associated with a monotonically decreasing risk of developing all the studied mental disorders except bipolar disorder. The association held even when comparing the cognitive abilities of brothers raised in the same family, attesting that cognitive ability and mental disorders are not associated because both arise from the same family background circumstances. Similarly, individuals with higher educational attainment had fewer mental health disorders. The association between low cognitive abilities and the risk of mental disorders was notably stronger in males with low educational attainment, compared to those with high educational attainment. These individuals may be an underutilized target group for mental-disorder prevention.

## Introduction

The association between higher educational attainment and better mental health is well established (Erickson et al., 2016), but the underlying mechanisms are complex and interwoven. The close association between cognitive abilities and educational attainment leaves an unexplored question about whether the link between low educational attainment and more mental health problems is better explained by low cognitive abilities. Improved understanding of the connection between cognitive ability and mental health is needed to inform prevention and treatment and to better address the needs of mental health patients who have low cognitive ability and low educational attainment.

Several studies suggest that high cognitive abilities predict fewer mental health symptoms and reduced likelihood of developing a mental disorder (Bridger & Daly, 2019; Gale et al., 2010; Jokela, 2022; Koenen et al., 2009; Lager et al., 2017; Mikkelsen et al., 2014; Mortensen et al., 2005; Navrady et al., 2017; Weiser et al., 2023; Williams et al., 2023; Wraw et al., 2016). These robust studies suggest that early measures of cognitive abilities can predict the development of later mental disorders. Similarly, several studies have shown that low educational-attainment levels predict more mental health problems (Boman, 2023; Demange et al., 2022; Erickson et al., 2016; Malanchini et al., 2019). Several studies have shown that the ability to obtain formal education is related to cognitive abilities. For example, Deary et al. (2007) found a correlation of .81 between cognitive ability measured at age 11 and school grades measured at age 16. Noncognitive abilities such as self-regulation, attitudes, and motivation have also been shown to independently influence both educational achievement and attainment (Boman, 2023; Demange et al., 2022; Malanchini et al., 2019). The finding that educational attainment is not merely a reflection of cognitive abilities suggests that educational attainment itself could be used as a unique predictor of mental disorders.

Previous research has not examined cognitive abilities and educational attainment together, leaving it unclear whether their effects on mental disorders are interactive or additive. In addition, most studies draw on selected samples affected by healthy-volunteer bias (Brayne & Moffitt, 2022; Fry et al., 2017). Individuals with lower cognitive abilities, reduced educational attainment, and pronounced mental health issues are less likely to participate and more likely to discontinue their involvement in longitudinal studies. Population-based studies are therefore particularly valuable. This article aims to assess three distinct hypotheses, using administrative data for the Norwegian male population.

### Hypothesis 1: Higher cognitive abilities are associated with a reduced risk of mental disorders, with a possible increased risk at very high levels of cognitive abilities

Although many studies report a negative correlation between cognitive abilities and mental health, some speculate that individuals with very high cognitive abilities might be susceptible to mental disorders because of a heightened sensitivity to the environment. For instance, Penney et al. (2015) found that undergraduates with higher intelligence showed more depressive rumination and worry. Similarly, Karpinski et al. (2018) surveyed American Mensa members—people who scored above the 98th percentile on IQ tests—and found that more individuals had self-reported mental disorders than expected, given national averages. This finding has been interpreted to support *hyperbrain theory*, which suggests that individuals with very high levels of cognitive ability have central nervous systems that are unable to properly relax.

### Hypothesis 2: There is a nonadditive relationship between cognitive abilities and educational attainment in predicting mental disorder

Prior research is limited by the lack of studies that consider cognitive abilities and educational attainment simultaneously. Consequently, it remains challenging to determine how these two closely related traits independently or interactively predict the onset of mental disorders. Specifically, the nature of their relationship—whether it involves interaction, dual primary effects, or suppression of one factor by the other—is not yet clearly understood. Only with both measures included is it possible to explore the relative contribution of each element.

### Hypothesis 3: Cognitive abilities predict the development of mental disorders above and beyond family factors

Mental health can be affected both by stable individual traits and by the surrounding environment, but the relative importance of traits versus environment is hard to disentangle. For example, one study found that the cognitive ability of an individual is a much stronger predictor of educational performance than parental occupational class, income, or education (O’Connell & Marks, 2021). We tested whether this also applies to mental disorders, hypothesizing that cognitive ability affects the probability of receiving a mental-disorder diagnosis independently of socioeconomic background. Parental education and income do not cover all socioeconomic aspects, so we compared brothers to control for shared familial factors. This approach reduces residual confounding (Sjölander et al., 2022; Taylor, 2021).

## Research Transparency Statement

### General disclosures

**Conflict of interest:** All authors declare no conflicts of interest. **Funding:** This work is part of the REMENTA project and was supported by the Research Council of Norway (No. 300668). This work was partly supported by the Research Council of Norway through its Centers of Excellence funding scheme, Project No. 262700. Additional support was provided by grants from the U.S. National Institute on Aging (P30AG034424) and the U.S. National Institute of Child Health and Human Development (P2C-HD065563). **Artificial intelligence:** Artificial intelligence (ChatGPT-4o) was used for proofreading the manuscript and for R code annotation. No other artificial-intelligence-assisted technologies were used in this research or the creation of this article. **Ethics:** The study was approved by the Regional Committee for Medical and Health Research Ethics. Because the data for this study came from deidentified administrative registers that Statistics Norway makes available for research at approved institutions, individual informed consent was not required.

### Study disclosures

**Fig. 1.**
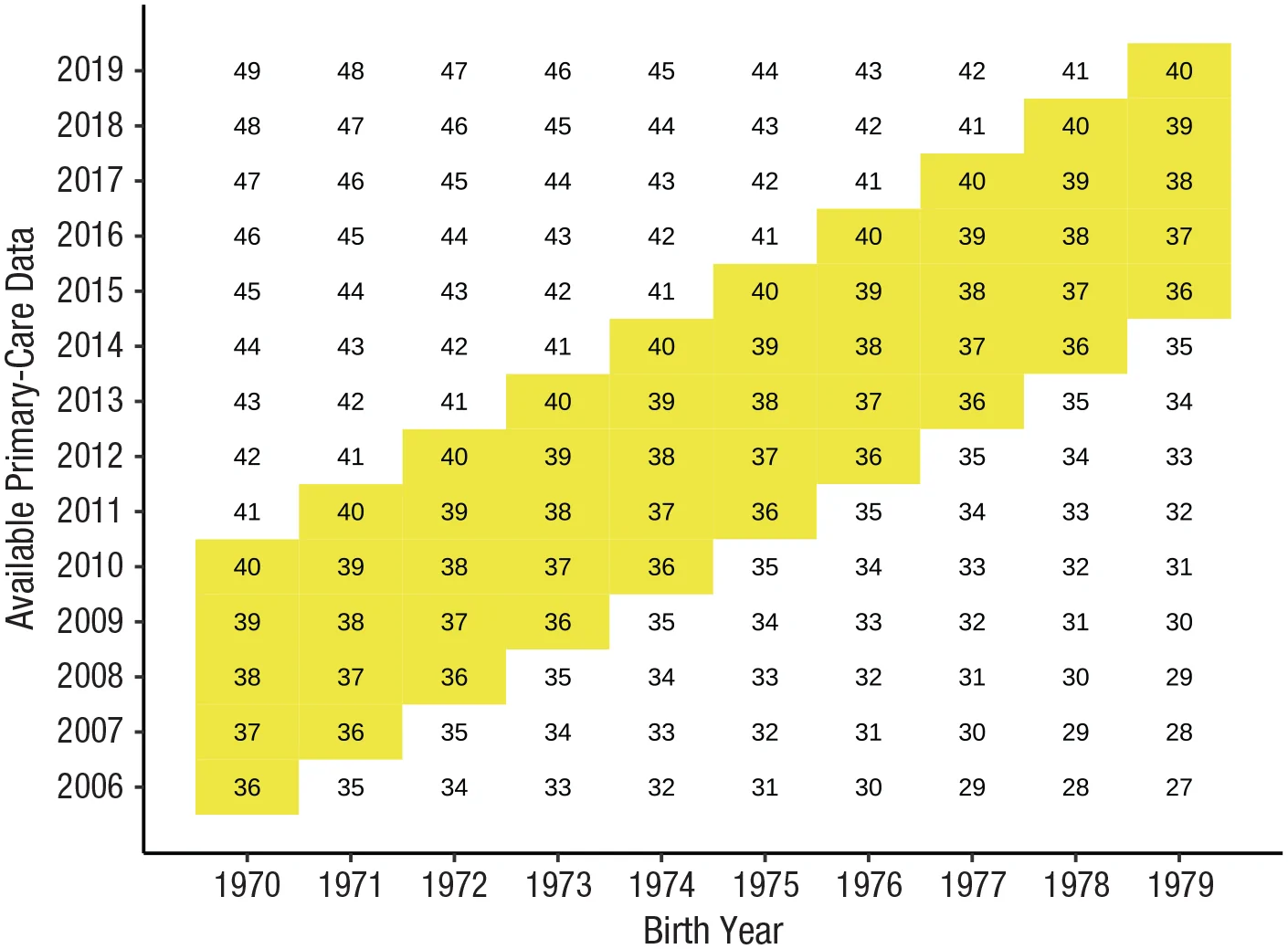
Study design: age of included birth cohorts and years of primary-care data on mental disorders.

**Fig. 2.**
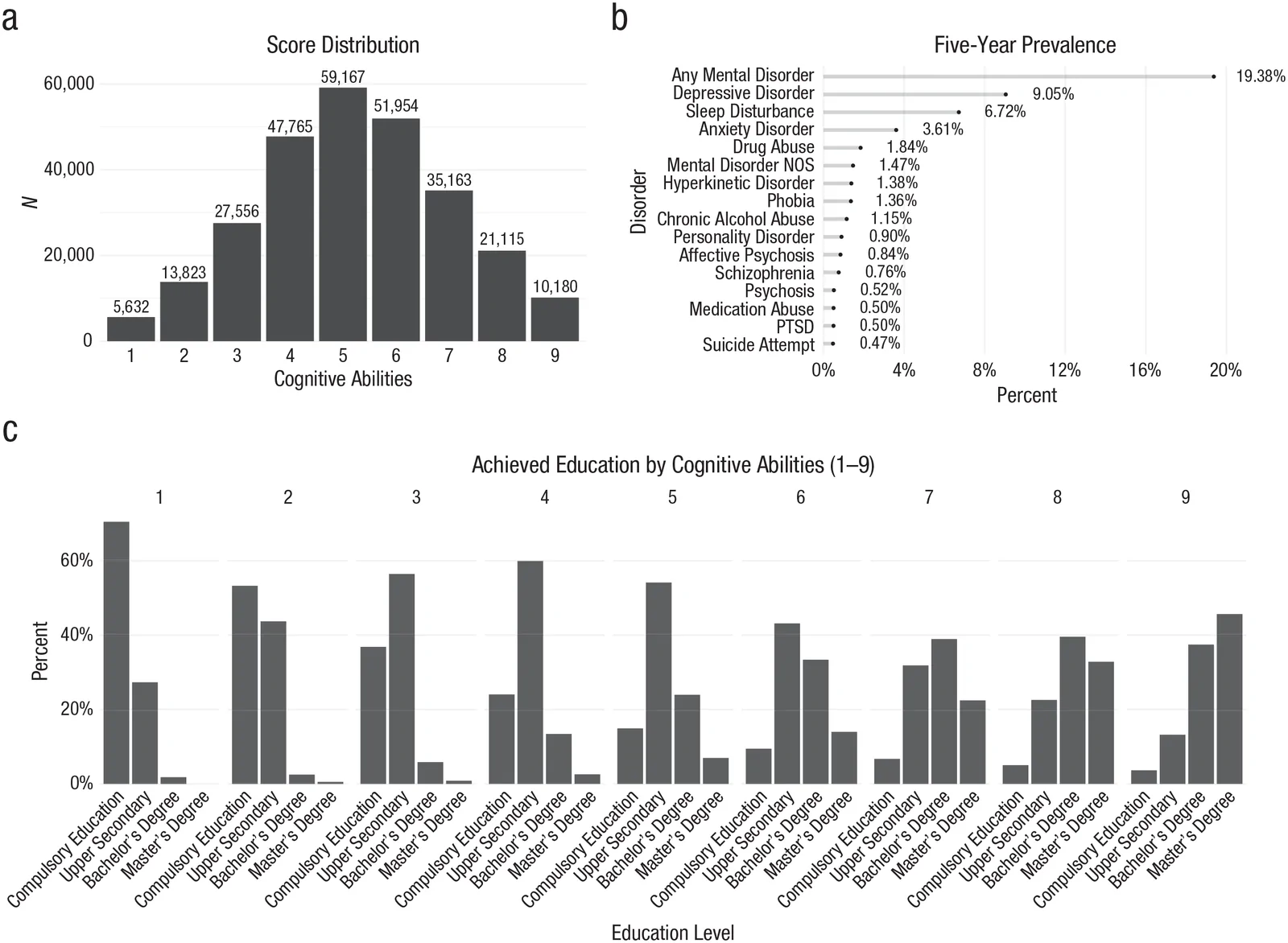
Summary of data. In (a) we show how cognitive abilities are distributed in the population; in (b) we show the prevalence rates for five mental disorders. In (c), we illustrate the proportion of individuals achieving various levels of educational attainment across the nine levels of cognitive abilities. NOS = not otherwise specified; PTSD = post-traumatic stress disorder.

**Fig. 3.**
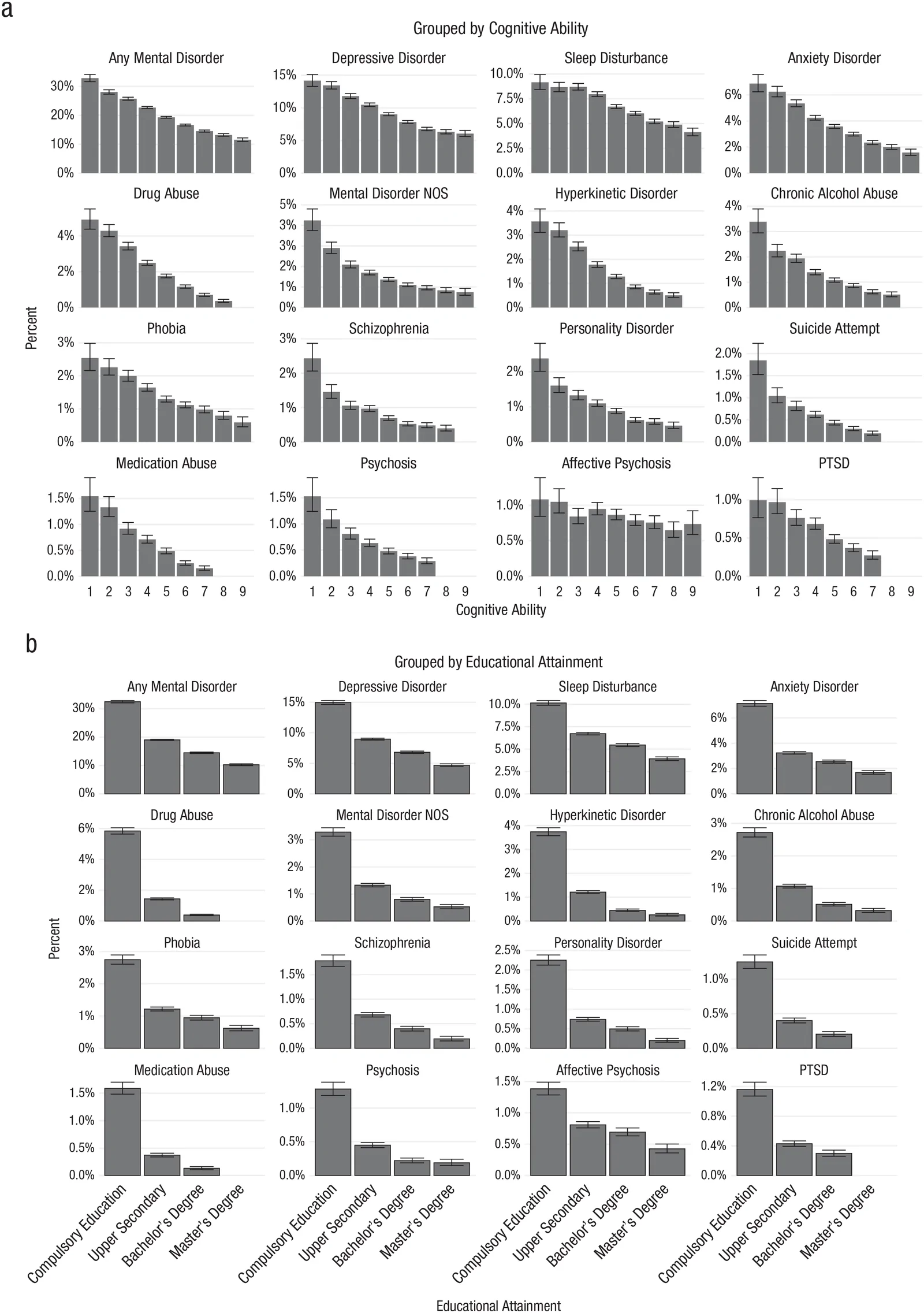
The average 5-year prevalence of adult mental disorders, grouped by level of cognitive ability (a) and educational attainment (b). The confidence intervals were calculated using the binomial proportion confidence interval; groups with fewer than 50 individuals were excluded. NOS = not otherwise specified; PTSD = post-traumatic stress disorder.

**Fig. 4.**
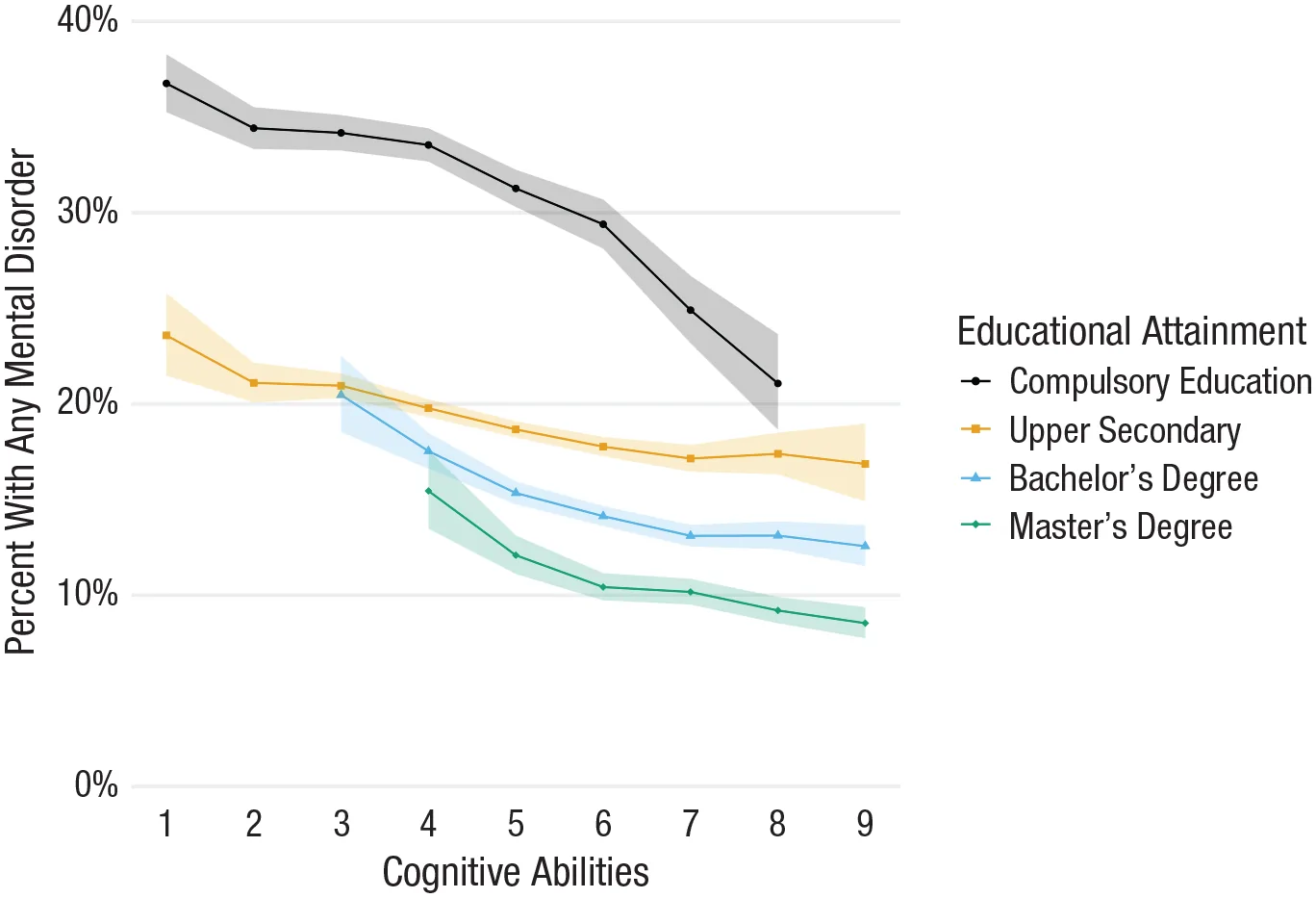
The average prevalence of adult mental disorders by cognitive abilities and educational attainment in combination. Confidence intervals (i.e., shaded areas) were calculated using the binomial proportion confidence interval; groups with fewer than 50 individuals were excluded.

**Preregistration:** This study was not preregistered. **Materials:** We do not have access to the Norwegian Armed Forces General Mental Ability Test Battery. Interested parties must apply directly to the Norwegian Armed Forces to request access. However, access to these materials is highly restricted because of their sensitive nature. The test battery is considered part of the Norwegian Armed Forces’ internal assessment tools, and its disclosure is limited in accordance with national defense and security considerations. Consequently, it is not publicly available, and it is unlikely that external researchers will be granted access. **Data:** The raw primary data cannot be made publicly available because they contain sensitive information. The data are controlled by third parties (Statistics Norway, Norwegian Armed Forces, and the Norwegian Regional Committees for Medical and Health Research Ethics), which allow restricted access. For details, please see their respective websites and our research transparency document (http://www.doi.org/10.17605/OSF.IO/YD3S8). Processed, summary-level data have been made publicly available at our Open Science Framework (OSF) repository (http://www.doi.org/10.17605/OSF.IO/GCTDJ). **Analysis scripts:** All analysis scripts are publicly available in the OSF repository (http://www.doi.org/10.17605/OSF.IO/GCTDJ). The OSF repository also includes the necessary .csv files required to reproduce the figures presented in the study. **Computational reproducibility:** Because the primary data cannot be shared, the reproducibility of this manuscript was not verified by the journal’s STAR team. We have made the R code for this project, including the analysis scripts and figure generation, available at the Open Science Framework (http://www.doi.org/10.17605/OSF.IO/GCTDJ). All figures are reproducible using summarized data. Due to the sensitivity and legal status of register data, we are unable to upload raw data. In addition to summarized data, we have provided a means to simulate a similar data set using the covariances and means from the original data set. The simulated data serve as a template for running our model scripts. Please note that this method is too coarse to replicate our results exactly. We have provided a quarto document that allows other parties to recreate Figures 1 to 4.

## Method

### Population sample

We used data from Norwegian conscripts born between 1970 and 1979 (*N* = 272,351). These 10 annual cohorts were chosen because of their high army-intake ratio. During years when these cohorts reached conscript age, the army-recruitment procedure was compulsory for all men and stringently enforced. Among males born in Norway who were born during this period and who were alive to participate, 95.9% $\left(\frac{272,351}{284,106}\right)$ attended the selection procedure. These cohorts also allowed us to ascertain mental-disorder outcomes using the Norwegian primary-care register, which was available only from 2006 to 2019, when our sample was 36 to 40 years old. The study design is shown in Figure 1.

### Sibling sample

The goal of the sibling analysis is to account for unobserved environmental effects that are shared between brothers by isolating the within-family variance of cognitive abilities. We identified males with at least one full brother who had participated in the armed forces recruitment procedure and who was also born between 1970 and 1979 (*N* = 87,583). Using the Norwegian registers, we identified brothers as individuals who shared the same mother and father ID markers.

### Adolescent cognitive abilities

Cognitive abilities were measured with the Norwegian Armed Forces general mental ability battery of tests, which is used for selection and classification. Most of the sample undertook the test the year they turned 18 years old (89%). The average testing age was 17.8 years (*SD* = 0.8). Before data release, each individual’s three subtest scores were standardized and averaged, resulting in a single integer score, which in turn was converted to a *stanine category* (STAndard NINE; 1–9) with a mean of 5 and a standard deviation of 2. Thus, a stanine score of 5 represents an IQ score of 100, and every stanine increment or decrement represents an increase or decrease of 7.5 IQ points. We defined exceptionally high cognitive abilities as a stanine score of 9, corresponding to scores above the 96th percentile. We use the term “cognitive abilities” instead of “intelligence” because the analyses did not include modeling of intelligence as a latent g factor (see Revelle, 2024, for a discussion).

We did not have access to the raw cognitive-ability data for this study; we had access only to the stanine scores provided by the Norwegian Armed Forces. The cognitive-abilities measure combines three subtests: progressive matrices, numerical reasoning, and word comprehension. The matrices, which were designed to be similar to those in the Wechsler Adult Intelligence Scale (WAIS) and Raven’s Standard Progressive Matrices, consist of 36 items with a time limit of 20 min. The word-comprehension test consists of 54 items with an 8-min time limit. Finally, the numerical-reasoning test consists of 30 items with a 25-min time limit. The composite measure is designed to include both fluid and knowledge components and has a correlation with the WAIS IQ test of 0.73 (Sundet et al., 1988). The Norwegian Armed Forces estimated the reliability of the composite at 0.77 using Raykov’s (2004) reliability coefficient. Raykov’s coefficient was used to account for differing item variances across the three subtests. This ensures that larger subtests, which have more items, do not disproportionately affect reliability. All tests were completed in a proctored environment. We excluded 3.8% of the sample because they lacked a recorded numerical cognitive score, for reasons such as very poor Norwegian language skills, suspected faking of bad performance (e.g., to get out of military service), or other issues with test administration.

### Educational attainment

We used Norwegian registry data to determine each male’s educational attainment by the age of 35 according to the International Standard Classification of Education’s four main brackets: compulsory education (1), post-secondary education (2), bachelor’s degree (3), and master’s degree or higher (4). Category 4 included PhDs $\left(0.8\%=\frac{2,168}{272,351}\right)$ . Educational attainment at age 35 closely mirrors lifetime achieved education, because very few Norwegians complete further formal education after age 35.

### Mental disorders

To ascertain diagnoses of mental disorders, we used the national register data set for reimbursement of primary care physicians (KUHR; *Kontroll og utbetaling av helserefusjoner* [control and payment of health reimbursements]), which covers information from 2006 to 2019, when our sample was 36 to 40 years old. All individuals who legally reside in Norway are assigned a general practitioner. To receive reimbursements, general practitioners send billing information to the Norwegian Health Economics Administration, including a diagnosis or reason for the visit. Since many patients with mental disorders require medication, sick leave, or further referral—all of which typically begin with a general-practitioner visit—it is unlikely that such visits go unreported in KUHR. Diagnostic information is coded according to the International Classification of Primary Care, 2nd edition (ICPC-2), and is registered in a database. We extracted all general-practitioner visits with valid diagnostic codes. We defined “mental disorder” as a visit recorded with an ICPC-2 mental-disorder diagnosis, ranging from P70 to P99. Note that P73, Affective Psychosis, is used instead of mania or bipolar disorders in ICPC-2. We also included P06 (sleep disturbance), P15 (chronic alcohol abuse), and P19 (drug abuse) in our analyses. Although the latter three codes are listed as “symptom” rather than “disorder” codes in ICPC-2, they capture important clinical features that are not captured in any of the available diagnostic diagnoses. We also included a summary variable, an Any Mental Disorder diagnosis, if an individual qualified for at least one of the included diagnoses between 36 and 40 years old. ICPC-2 diagnoses primarily stem from general-practitioner visits (95.3%), whereas a smaller proportion (4.7%) originate from consultations with a medical doctor in the emergency room. See the supplemental files in the Supplemental Material available online for an overview of the ICPC codes and corresponding ICD10 diagnoses.

### Parental socioeconomic status

Data on lifetime educational attainment and personal income were available for parents of the men from Statistics Norway. Parents’ educational attainment was also coded according to the International Standard Classification of Education brackets: compulsory (1), post-secondary (2), bachelor’s degree (3), and master’s degree or higher (4). Parental income was the sum of the mother’s and father’s income during the birth year for each male. We standardized income by offspring birth year and converted these scores into percentiles to account for inflation.

### Statistical procedure

Our first step was to assess the relationship between cognitive abilities and mental disorders. Plotting the proportion who experience a mental disorder with respect to each stanine level gives a visual tool to assess how cognitive abilities are associated with mental disorders. We also contrasted the fit of linear and curvilinear models to formally assess the relationship, presented in the appendix in the Supplemental Material. We further applied linear models to explore the relationship between cognitive abilities, socioeconomic status, educational attainment, and mental disorders. All our models are logistic regressions using the *glm* function with mental disorder dummy-coded as 0 (no diagnosis) or 1 (diagnosis). Cognitive ability (CA) level, educational attainment and income were treated as continuous variables. We began with an unadjusted model (Model 1):

(1) $$logit(P(Y=1))=\beta_{0}+\beta_{1}.\mathrm{CA}.$$

Next, in Model 2 we added parental socioeconomic status, defined as parental educational attainment and income:

(2) $$\begin{array}{l} logit(P(Y=1))=\beta_{0}+\beta_{1}.\;\mathrm{CA}+\beta_{2}.MotherEducation\\ \;\;+\beta_{3}.FatherEducation+\beta_{4}.\mathrm{Income}. \end{array}$$

In Model 3, we also added each male’s own educational attainment to separate between the effects of cognitive abilities and educational attainment:

(3) $$\begin{array}{l} logit(P(Y=1))=\beta_{0}+\beta_{1}.\;\mathrm{CA}+\beta_{2}.MotherEducation\\ \;\;+\beta_{3}.FatherEducation+\beta_{4}.\mathrm{Income}\\ \;\;+\beta_{5}.\mathrm{EducationalAttainment}. \end{array}$$

Last, in Model 4 we compared brothers by leveraging within-family (WF) variation across all model variables. Specifically, we employed a fixed-effect logistic regression model in which each variable is expressed as its deviation from the family mean:

(4) $$\begin{array}{l} logit(P(Y^{WF}=1))=\beta_{0}+\beta_{1}{\mathrm{.CA}}_{WF}\\ \;\;+\beta_{2}{\mathrm{.EducationalAttainment}}_{WF}. \end{array}$$

This procedure adjusts by design for confounding factors that influence the cognitive abilities of all brothers in a family; for most full brothers this includes social influences, such as growing up in the same family and in the same household, as well as 50% of genetic variation. (See McNeish, 2023, for an introduction to fixed-effect models in psychology.) The files in the Supplemental Material include additional models that directly test some of the results from the article. We used R (Version 4.3.0) for data management and modeling. We excluded all information for identified subgroups of fewer than 50 individuals in order to ensure privacy.

## Results

### Distribution of cognitive ability, educational attainment, and mental disorders

As shown in Figure 2a, the cognitive-ability composite approximated a normal distribution with only slight skewness toward the above-average scores. Figure 2b shows the 5-year prevalence rate for each disorder between the ages of 36 and 40 years. A large minority of males received a mental-disorder diagnosis (19.38%). The most prevalent disorders were depressive disorder (9.1%), sleep disturbance (6.7%), and anxiety disorder (3.6%). Figure 2c shows a bar graph of the proportion of individuals in each cognitive-ability stanine group and their educational attainment. As expected, although individuals with scores in the lower end of the cognitive-ability measure rarely attained bachelor’s or master’s degrees, individuals with scores in the higher end typically held such qualifications.

### Hypothesis 1: Results

Figure 3 shows a breakdown of the proportion of adults with a given mental-disorder diagnosis grouped by cognitive abilities (a) and educational attainment (b). We calculated the proportion as $\frac{Individuals\;with\;disorder\;in\;the\;cognitive-abilities\;group}{All\;individuals\;in\;the\;respective\;cognitive-abilities\;group}$ . We found a monotonic relationship between cognitive abilities and the likelihood of receiving a mental-disorder diagnosis. When comparing the top and bottom cognitive-abilities groups, there was a threefold increase—from 10% receiving any mental-disorder diagnosis in stanine 9 (top) to 30% receiving any mental-disorder diagnosis in stanine 1 (bottom). The one exception is for affective psychosis, for which we did not find a strong association. Our results show that higher levels of educational attainment were associated with a decreased risk of developing mental disorders. Of special note, the group with only compulsory education stood out with a particularly high risk for every disorder. Our data were not consistent with the hypothesis that individuals with exceptionally high cognitive abilities experience more mental disorders. For all high-prevalence disorders, men with the highest possible stanine had the lowest likelihood of receiving a mental-disorder diagnosis. Rare disorders were more susceptible to statistical fluctuations, but no disorder clearly indicated a higher risk of diagnosis in individuals with exceptionally high cognitive abilities.

### Hypothesis 2: Results

We also assessed the relation between cognitive abilities, educational attainment, and any mental disorder by plotting the percentage of men who were given a diagnosis in each group (defined by cognitive ability and educational attainment; see Fig. 4). We excluded groups with fewer than 100 men to remove potentially unreliable results. These results showed that cognitive ability is clearly associated with mental disorders above and beyond educational attainment. But in addition, mental disorders were most prevalent among males with only a compulsory education. Men with the combination of low cognitive abilities and compulsory educational attainment were most likely to receive a mental-disorder diagnosis. The difference between the groups with the highest risk (low cognitive abilities and compulsory education: 37.8%) and lowest risk (high cognitive abilities and a master’s degree: 8.5%) was 29.3 percentage points.

### Hypothesis 3: Results

**Fig. 5.**
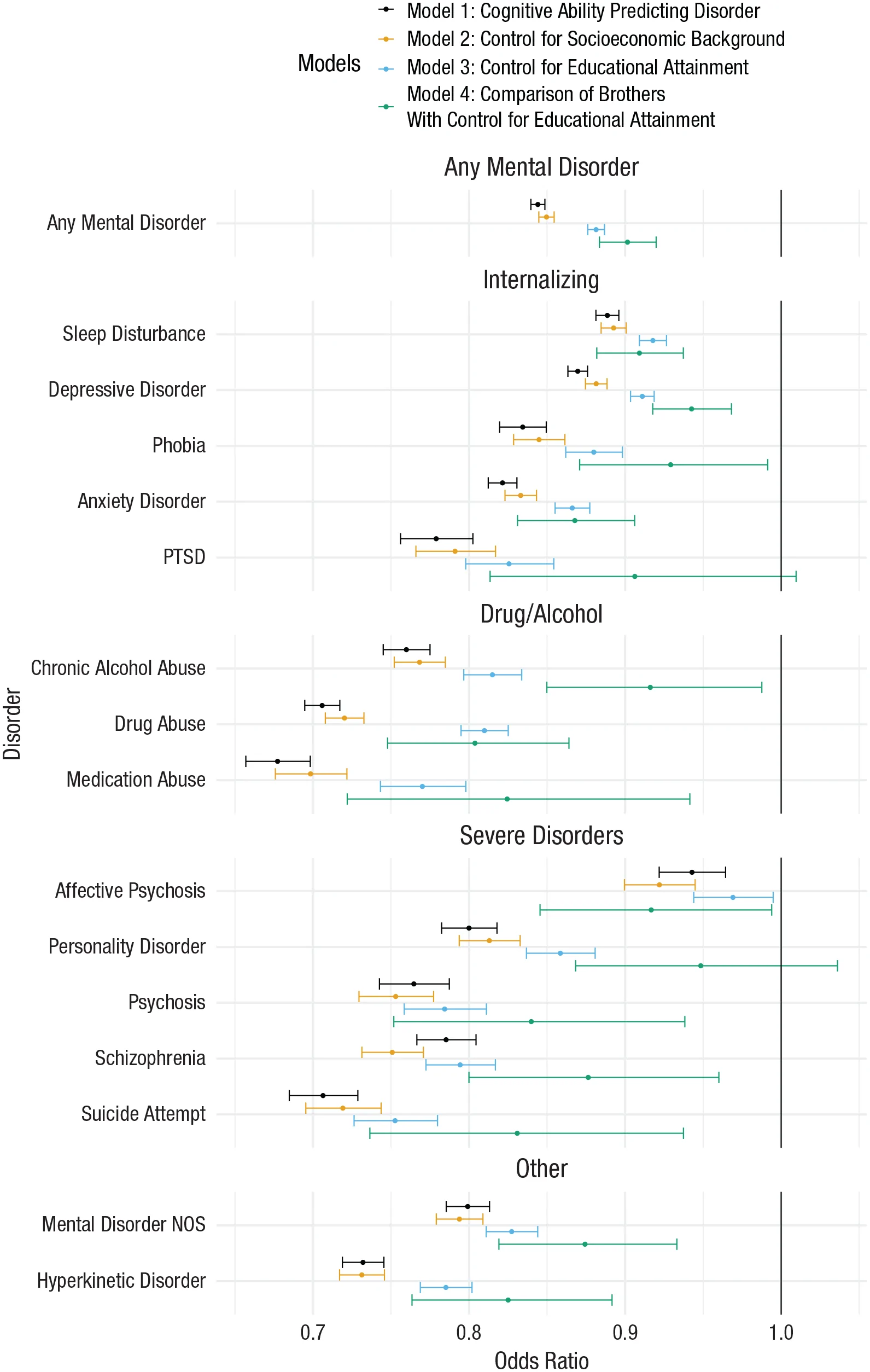
The odds ratio for receiving a mental-disorder diagnosis given a 1-point increase in cognitive ability. Cognitive abilities are measured in integers from 1 (lowest) to 9 (highest) in stanines. NOS = not otherwise specified; PTSD = post-traumatic stress disorder. The plotted coefficient from cognitive abilities. 95% confidence interval.

See Figure 5 for the coefficients describing the relationship between adolescent cognitive abilities and adult mental disorders for each of our four statistical models. The *x*-axis represents the odds ratio (*OR*) for each diagnosis. There was a pronounced relation between cognitive abilities and the likelihood of receiving a primary-care mental-disorder diagnosis (Model 1). This relation is not accounted for by parental socioeconomic factors (Model 2). Introducing the effect of educational attainment diminished the association for all disorders (Model 3), especially alcohol and drug-use disorders. The sibling-comparison analysis showed a heightened risk of receiving a mental-disorder diagnosis for individuals with lower cognitive abilities, although the effect size was attenuated within the pool of brothers (Model 4). Notably, the occurrence of both post-traumatic stress disorder (PTSD) and personality disorder were no longer statistically significant. None of our models had a variance-inflation factor above 1.4. See Table 1 for model summary statistics.

**Table 1.** Model Estimates Across Four Models.

Outcome	Model 1					Model 2					Model 3					Model 4				
	Coef	95% CI	*SE*	*p*	*R*²	Coef	95% CI	*SE*	*p*	*R*²	Coef	95% CI	*SE*	*p*	*R*²	Coef	95% CI	*SE*	*p*	*R*²
Any mental disorder																				
Any mental disorder	0.84	[0.84, 0.85]	1.00	< .001	.02	0.85	[0.84, 0.85]	1.00	< .001	.02	0.88	[0.88, 0.89]	1.00	< .001	.03	0.90	[0.88, 0.92]	1.01	< .001	.03
Internalizing																				
Anxiety disorder	0.82	[0.81, 0.83]	1.01	< .001	.02	0.83	[0.82, 0.84]	1.01	< .001	.02	0.87	[0.86, 0.88]	1.01	< .001	.02	0.87	[0.83, 0.91]	1.02	< .001	.03
Depressive disorder	0.87	[0.86, 0.88]	1.00	< .001	.01	0.88	[0.87, 0.89]	1.00	< .001	.01	0.91	[0.90, 0.92]	1.00	< .001	.02	0.94	[0.92, 0.97]	1.01	< .001	.02
Sleep disturbance	0.89	[0.88, 0.90]	1.00	< .001	.01	0.89	[0.88, 0.90]	1.00	< .001	.01	0.92	[0.91, 0.93]	1.00	< .001	.01	0.91	[0.88, 0.94]	1.02	< .001	.02
PTSD	0.78	[0.76, 0.80]	1.02	< .001	.02	0.79	[0.77, 0.82]	1.02	< .001	.02	0.83	[0.80, 0.85]	1.02	< .001	.02	0.91	[0.81, 1.01]	1.06	.074	.05
Phobia	0.83	[0.82, 0.85]	1.01	< .001	.01	0.84	[0.83, 0.86]	1.01	< .001	.01	0.88	[0.86, 0.90]	1.01	< .001	.02	0.93	[0.87, 0.99]	1.03	.026	.04
Severe disorders																				
Personality disorder	0.80	[0.78, 0.82]	1.01	< .001	.01	0.81	[0.79, 0.83]	1.01	< .001	.02	0.86	[0.84, 0.88]	1.01	< .001	.02	0.95	[0.87, 1.04]	1.05	.241	.08
Affective psychosis	0.94	[0.92, 0.96]	1.01	< .001	.00	0.92	[0.90, 0.94]	1.01	< .001	.00	0.97	[0.94, 0.99]	1.01	.019	.01	0.92	[0.85, 0.99]	1.04	.035	.03
Psychosis	0.76	[0.74, 0.79]	1.02	< .001	.02	0.75	[0.73, 0.78]	1.02	< .001	.02	0.78	[0.76, 0.81]	1.02	< .001	.02	0.84	[0.75, 0.94]	1.06	.002	.12
Schizophrenia	0.79	[0.77, 0.80]	1.01	< .001	.02	0.75	[0.73, 0.77]	1.01	< .001	.02	0.79	[0.77, 0.82]	1.01	< .001	.03	0.88	[0.80, 0.96]	1.05	.005	.13
Suicide attempt	0.71	[0.68, 0.73]	1.02	< .001	.03	0.72	[0.70, 0.74]	1.02	< .001	.03	0.75	[0.73, 0.78]	1.02	< .001	.04	0.83	[0.74, 0.94]	1.06	.003	.08
Other																				
Hyperkinetic disorder	0.73	[0.72, 0.75]	1.01	< .001	.03	0.73	[0.72, 0.75]	1.01	< .001	.03	0.79	[0.77, 0.80]	1.01	< .001	.05	0.83	[0.76, 0.89]	1.04	< .001	.15
Mental disorder NOS	0.80	[0.79, 0.81]	1.01	< .001	.02	0.79	[0.78, 0.81]	1.01	< .001	.02	0.83	[0.81, 0.84]	1.01	< .001	.02	0.87	[0.82, 0.93]	1.03	< .001	.07
Drug/Alcohol																				
Chronic alcohol abuse	0.76	[0.75, 0.77]	1.01	< .001	.02	0.77	[0.75, 0.78]	1.01	< .001	.02	0.81	[0.80, 0.83]	1.01	< .001	.03	0.92	[0.85, 0.99]	1.04	.022	.07
Medication abuse	0.68	[0.66, 0.70]	1.02	< .001	.04	0.70	[0.68, 0.72]	1.02	< .001	.04	0.77	[0.74, 0.80]	1.02	< .001	.06	0.82	[0.72, 0.94]	1.07	.004	.16
Drug abuse	0.71	[0.69, 0.72]	1.01	< .001	.04	0.72	[0.71, 0.73]	1.01	< .001	.04	0.81	[0.79, 0.83]	1.01	< .001	.08	0.80	[0.75, 0.86]	1.04	< .001	.18

Note: CI = confidence interval; NOS = not otherwise specified; coeff = coefficient; PTSD = post-traumatic stress disorder.

## Discussion

This study adds to our current understanding of the link between cognitive ability and mental disorder in several ways. First, higher cognitive abilities are associated with a monotonically decreasing risk of presenting with a mental disorder in primary care among adult males. The association between cognitive abilities and mental-disorder diagnosis is strong for all disorders except for affective psychosis (bipolar disorder, mania). There was no indication that individuals with extremely high cognitive abilities had an increased likelihood of receiving a mental-disorder diagnosis. Secondly, the association between low cognitive abilities and elevated risk of mental disorders was notably stronger in males who also had low educational attainment, compared with males with high educational attainment. Third, the association between cognitive abilities and mental disorder held even when comparing the cognitive ability of brothers within a family, and thus it is not likely to be caused by family background circumstances.

### Conclusions

Our results mirror the findings of previous studies indicating that low cognitive abilities are associated with greater risk of mental disorders and that low educational attainment is associated with greater risk of mental disorder. The various mental disorders differed in the strength of the association with cognitive abilities. It was weaker for internalizing disorders compared with those typically classified as externalizing or thought disorders. These observations raise an important question about the underlying mechanisms—whether the connection between cognitive ability and mental disorders is due to common factors across all conditions or whether different factors lead to similar outcomes. Previous research has highlighted multiple potential pathways through which cognitive abilities may influence mental health and well-being (Fuhrmann et al., 2022). Our findings underscore the need for further investigation into the distinct reasons that may contribute to the association between cognitive ability and disorder. Consistent with previous studies (Koenen et al., 2009; Smith et al., 2015; Sniekers et al., 2017), we find that affective psychosis (bipolar disorder) stands out from the rest of the disorders. Whereas all the other diagnoses show a clear association with low cognitive abilities, this relation is noisier for affective psychosis.

Cognitive abilities may be linked to mental disorders for multiple reasons, which are not mutually exclusive. One possibility is that persistent stress stemming from underachievement might precipitate mental disorders. A complementary explanation is that individuals with lower cognitive abilities often attain less education, leading to lower-paying occupations, and hence may find themselves working in suboptimal conditions and residing in low-income neighborhoods. This scenario increases their exposure to multiple risk factors for mental disorders, both in their work and home environments. Another possibility is inadequate treatment for mental health problems in individuals with low cognitive ability (Harvey et al., 2014; Knekt et al., 2014). It might be that life is particularly stressful for individuals who have low cognitive abilities and who also fail to obtain a degree or an apprenticeship certification.

Contrary to previous reports (Karpinski et al., 2018; Penney et al., 2015), we did not find that individuals with very high cognitive abilities had an increased likelihood of receiving a mental-disorder diagnosis. Prior studies suggesting that high cognitive abilities negatively affect mental health are likely to have been influenced by nonrepresentative samples, leading to confounded outcomes. Although there is strong evidence for the link between high cognitive abilities and reduced risk of mental disorders, diverging subpopulation patterns can be masked by global averages. For example, research shows that individuals who score higher on the autism spectrum are vulnerable to mental disorders and show greater variability in cognitive abilities, leading to a greater proportion of extremely gifted people compared with the population (Billeiter & Froiland, 2023).

The strong association between cognitive ability and mental disorder, even when isolating the variation in cognitive abilities among brothers within families, indicates that this link cannot be fully explained by family background, social class, or other factors that equally affect siblings. We interpret these findings to suggest that only a portion of the correlation between cognitive ability and mental disorders is attributable to educational attainment. This aligns with research findings that suggest that the association between life outcomes and cognitive abilities is not inherently due to socioeconomic factors, such as income and social class (Jokela, 2022; O’Connell & Marks, 2021). The capacity of within-family models to control for social confounding relies on brothers having lived together in the same family, which applies to most, but not all, brothers.

### Strengths and limitations

Our investigation goes beyond the scope of prior research by incorporating a population-wide primary-care data set alongside a high-quality measure of cognitive ability. Given the wide coverage and frequent use of Norwegian primary care, we have low risk of either participation bias or bias due to missing data. Caspi et al. (2024) found that 99.1% of the Norwegian population consulted their general practitioner between 2006 and 2019, underscoring the extensive reach of primary care.

The study also has some weaknesses. First, relying on primary-care diagnoses means that individuals who do not seek treatment or sick leave are misclassified. We cannot rule out potential bias if individuals with higher cognitive abilities are less likely to visit their general practitioner. Second, we were able to evaluate only diagnoses between the age of 36 and 40 for Norwegian males. Given Norway’s universal health-care system and its social welfare policies, findings from this context may not generalize to other regions. Norway’s robust social-support systems and egalitarian society can influence both the likelihood of diagnosis and overall health outcomes, potentially leading to distinct patterns that may not replicate in other settings. Third, although the prospective nature of our data allowed us to probe the causal implications of cognitive abilities and educational attainment for mental disorders, we cannot rule out possible back-door causal pathways. For instance, mental health problems in adolescence could influence both educational attainment and adult mental health.

Considering our findings and previous literature, it appears that people with lower cognitive abilities are more vulnerable to a wide range of mental disorders. Numerous studies indicate that low cognitive abilities precede the diagnosis of a mental disorder, supporting the argument for a causal impact. However, the present research cannot conclusively determine whether early-life mental distress or disorders influence cognitive abilities, leaving the possibility of reverse causality. We find that the relationship between cognitive abilities and mental disorders is present across the entire range of cognitive abilities and is consistent regardless of social background. Additionally, individuals with both low cognitive abilities and low educational attainment have an especially high likelihood of being diagnosed with a mental disorder in adulthood.

## Supplemental Material

sj-docx-1-pss-10.1177_09567976251347221 – Supplemental material for Cognitive Abilities and Educational Attainment as Antecedents of Mental Disorders: A Total Population Study of Males Supplemental material, sj-docx-1-pss-10.1177_09567976251347221 for Cognitive Abilities and Educational Attainment as Antecedents of Mental Disorders: A Total Population Study of Males by Magnus Nordmo, Hans Fredrik Sunde, Thomas H. Kleppestø, Morten Nordmo, Avshalom Caspi, Terrie E. Moffitt and Fartein Ask Torvik in Psychological Science

## Transparency

*Action Editor:* Tom Beckers

*Editor:* Simine Vazire

*Author Contributions*

**Magnus Nordmo:** Conceptualization; Data curation; Formal analysis; Investigation; Methodology; Project administration; Software; Visualization; Writing – original draft; Writing – review & editing.

**Hans Fredrik Sunde:** Conceptualization; Formal analysis; Methodology; Resources; Writing – original draft; Writing – review & editing.

**Thomas H. Kleppestø:** Conceptualization; Formal analysis; Investigation; Methodology; Writing – original draft; Writing – review & editing.

**Morten Nordmo:** Formal analysis; Methodology; Resources; Supervision; Writing – original draft; Writing – review & editing.

**Avshalom Caspi:** Conceptualization; Formal analysis; Investigation; Methodology; Supervision; Writing – original draft; Writing – review & editing.

**Terrie E. Moffitt:** Conceptualization; Investigation; Methodology; Supervision; Writing – original draft; Writing – review & editing.

**Fartein Ask Torvik:** Conceptualization; Formal analysis; Funding acquisition; Investigation; Methodology; Project administration; Resources; Software; Supervision; Visualization; Writing – original draft; Writing – review & editing.

**Artificial Intelligence:** Artificial intelligence (ChatGPT-4o) was used for proofreading the manuscript and for R code annotation. No other artificial-intelligence-assisted technologies were used in this research or the creation of this article.

**Ethics:** The study was approved by the Regional Committee for Medical and Health Research Ethics. Because the data for this study came from deidentified administrative registers that Statistics Norway makes available for research at approved institutions, individual informed consent was not required.

**Open Practices:** Preregistration: This study was not preregistered. Materials: We do not have access to the Norwegian Armed Forces General Mental Ability Test Battery. Interested parties must apply directly to the Norwegian Armed Forces to request access. However, access to these materials is highly restricted because of their sensitive nature. The test battery is considered part of the Norwegian Armed Forces’ internal assessment tools, and its disclosure is limited in accordance with national defense and security considerations. Consequently, it is not publicly available, and it is unlikely that external researchers will be granted access. Data: The raw primary data cannot be made publicly available because they contain sensitive information. The data are controlled by third parties (Statistics Norway, Norwegian Armed Forces, and the Norwegian Regional Committees for Medical and Health Research Ethics), which allow restricted access. For details, please see their respective websites and our research transparency document (http://www.doi.org/10.17605/OSF.IO/YD3S8). Processed, summary-level data have been made publicly available at our Open Science Framework (OSF) repository (http://www.doi.org/10.17605/OSF.IO/GCTDJ). Analysis scripts: All analysis scripts are publicly available in the OSF repository (http://www.doi.org/10.17605/OSF.IO/GCTDJ). The OSF repository also includes the necessary .csv files required to reproduce the figures presented in the study. Computational reproducibility: Because the primary data cannot be shared, the reproducibility of this manuscript was not verified by the journal’s STAR team. We have made the R code for this project, including the analysis scripts and figure generation, available at the Open Science Framework (http://www.doi.org/10.17605/OSF.IO/GCTDJ). All figures are reproducible using summarized data. Due to the sensitivity and legal status of register data, we are unable to upload raw data. In addition to summarized data, we have provided a means to simulate a similar data set using the covariances and means from the original data set. The simulated data serve as a template for running our model scripts. Please note that this method is too coarse to replicate our results exactly. We have provided a quarto document that allows other parties to recreate Figures 1 to 4.
