# Supplementary material for: Cognitive Abilities and Educational Attainment as Antecedents of Mental Disorders: A Total Population Study of Males
Source: Psychol Sci. 2025 Jun 26;36(7):499–513. doi: 10.1177/09567976251347221 (PMC12335617; doi:10.1177/09567976251347221)
Supplement: sj-docx-1-pss-10.1177_09567976251347221 – Supplemental material for Cognitive Abilities and Educational Attainment as Antecedents of Mental Disorders: A Total Population Study of Males [file sj-docx-1-pss-10.1177_09567976251347221.docx]

Supplemental Material

Content

[Are the relationships between cognitive abilities and mental disorders nonlinear? 2](#_Toc184192800)

[Supplemental Table 1: Linear Compared to Quadratic Models 2](#_Toc184192801)

[Is there a statistically significant interaction effect between cognitive abilities and educational attainment, when predicting mental disorder? 2](#_Toc184192802)

[Supplemental Table 2: Cognitive Abilities and Educational Attainment Interaction 3](#_Toc184192803)

[Supplemental Table 3: Ordinal Interaction 4](#_Toc184192804)

[Table of regression model coefficients 5](#_Toc184192805)

[Supplemental Table 4: Unadjusted 5](#_Toc184192806)

[Supplemental Table 5: Adjusted for Parental Education and Income 6](#_Toc184192807)

[Supplemental Table 6: Adjusted for Parental Education, Income and Educational Attainment 7](#_Toc184192808)

[Supplemental Table 7: Within-Family Cognitive Abilities 8](#_Toc184192809)

[Logistic regression while treating cognitive abilities as an ordinal variable 9](#_Toc184192810)

[Supplemental Table 8: Unadjusted Model 10](#_Toc184192811)

[Supplemental Figure 1: Unadjusted Model 11](#_Toc184192812)

[Supplemental Table 9: Adjusted for Parental Education and Income 12](#_Toc184192813)

[Supplemental Figure 2: Ordinal Model Adjusted for Parental Education and Income 13](#_Toc184192814)

[Supplemental Table 10: Adjusted for Parental Education, Income and Educational Attainment 14](#_Toc184192815)

[Supplemental Figure 3: Ordinal Model Adjusted for Parental Education, Income and Educational Attainment 15](#_Toc184192816)

[Supplemental Table 11: ICPC-2 Codes 16](#_Toc184192817)

## Are the relationships between cognitive abilities and mental disorders nonlinear?

One of the main goals of this paper is to assess the relationship between mental disorders and cognitive abilities assessed on a nine-point scale (hypothesis one). **Figure 3 Panel A** shows that the proportion who receive a mental disorder diagnosis decreases monotonically with increasing cognitive abilities. We also wanted to assess formal statistical models that are either linear or quadratic such that $disorder \sim cognitive$ or $disorder \sim cognitive+{cognitive}^{2}$. Disorder is a binomial variable while cognitive abilities is a continuous variable on a nine-point scale. The linear and quadratic models were then assessed using a likelihood ratio test. See the R code for more details. By contrasting the linear and quadratic curves, we find that sleep disturbance, medication abuse, drug abuse, schizophrenia, PTSD and mental disorder NOS show a statistically significant deviation. Note that the change in model fit from linear to quadratic is specified for both AIC and BIC.

| Supplemental Table 1: Linear Compared to Quadratic Models | | | | | |
| --- | --- | --- | --- | --- | --- |
| Disorder | P value | AIC Linear | AIC Quadratic Δ | BIC Linear | BIC Quadratic Δ |
| Any mental disorder | 0.051 | 263835.63 | -1.82 | 263856.66 | 8.7 |
| Sleep disturbance | 0.000* | 133401.38 | -10.93 | 133422.41 | -0.41 |
| Chronic alcohol abuse | 0.680 | 33511.80 | 1.83 | 33532.83 | 12.35 |
| Medication abuse | 0.000* | 16527.36 | -16.33 | 16548.39 | -5.82 |
| Drug abuse | 0.000* | 48147.72 | -49.84 | 48168.75 | -39.33 |
| Schizophrenia | 0.001* | 23869.32 | -8.48 | 23890.35 | 2.03 |
| Affective psychosis | 0.807 | 26433.88 | 1.94 | 26454.91 | 12.45 |
| Anxiety disorder | 0.151 | 83417.57 | -0.06 | 83438.60 | 10.45 |
| Depressive disorder | 0.174 | 163981.43 | 0.15 | 164002.46 | 10.67 |
| Suicide attempt | 0.708 | 15846.65 | 1.86 | 15867.68 | 12.38 |
| Phobia | 0.540 | 38845.79 | 1.63 | 38866.82 | 12.14 |
| Personality disorder | 0.083 | 27457.60 | -1.01 | 27478.62 | 9.81 |
| Hyperkinetic disorder | 0.212 | 38601.45 | 0.44 | 38622.48 | 10.95 |
| PTSD | 0.003* | 16813.99 | -6.91 | 16835.02 | -2.2 |
| Psychosis | 0.402 | 17319.53 | 1.3 | 17340.56 | 11.76 |
| Mental disorder NOS | 0.000* | 41036.42 | -21.27 | 41057.45 | -10.76 |

## Is there a statistically significant interaction effect between cognitive abilities and educational attainment, when predicting mental disorder?

We also formally assessed an interaction effect between cognitive abilities and educational attainment, predicting the development of a mental disorder. While **Figure 4** visually illustrated that the individuals with compulsory education and low cognitive abilities were most susceptible to developing a mental disorder, a regression model allows for hypothesis testing. We employed the following model $anydisorder \sim cognitiv+attainment +cognitiv*attainment.$ Any disorder is a binomial variable indicating whether the individual was diagnosed with any mental disorder the years between 36 and 40. Cognitive reflects cognitive abilities on a nine-point scale, while attainment is a modelled as a continuous variable of educational attainment using: 0 – Compulsory Education, 1 – Upper Secondary Education, 2 – Bachelors Degree, 3 – Masters Degree or higher.

| **Supplemental Table 2: Cognitive Abilities and Educational Attainment Interaction** | | | | |
| --- | --- | --- | --- | --- |
| Coefficient | Estimate | Std. Error | t value | P value |
| Intercept | 0.3827 | 0.0034 | 109.75 | < .001^***^ |
| CA | -0.0220 | 0.0007 | -30.67 | < .001^***^ |
| Educational Attainment | -0.1126 | 0.0027 | -41.34 | < .001^***^ |
| CA*Educational Attainment | 0.0094 | 0.0004 | 20.87 | < .001^***^ |

In addition, we ran the same model using educational attainment as an ordinal factor. Education was categorized into four levels: Compulsory education, upper secondary education, bachelor's degree, and master's degree or higher, with the latter serving as the reference category. The results show that the interaction term is statistically significant only for individuals with compulsory education, suggesting that the interaction effect is primarily driven by this group. Specifically, among individuals with only compulsory education, cognitive ability is a stronger predictor compared to the other levels of educational attainment.

| **Supplemental Table 3: Ordinal Interaction** | | | | |
| --- | --- | --- | --- | --- |
| **Coefficient** | **Estimate** | **Std. Error** | **t value** | **P value** |
| Intercept | 0.1671576 | 0.0105874 | 15.788 | < .001^***^ |
| CA | -0.0094045 | 0.0015103 | -6.227 | < .001^***^ |
| Bachelor’s degree | 0.0439186 | 0.0124118 | 3.538 | < .001^***^ |
| Upper Secondary | 0.0640658 | 0.0111889 | 5.726 | < .001^***^ |
| Compulsory Education | 0.2259960 | 0.0114164 | 19.796 | < .001^***^ |
| CA*Bachelor’s Degree | -0.0014116 | 0.0018273 | -0.773 | 0.439 |
| CA*Upper Secondary | 0.0009401 | 0.0016696 | 0.563 | 0.573 |
| CA*Compulsory Education | -0.0081154 | 0.0018117 | -4.480 | < .001^***^ |

## Table of regression model coefficients

The tables below show the coefficients from each respective regression model, while standard errors are shown in parenthesis. The comparisons of brothers models utilize clustered standard errors to account for family clustering.

Note that three *** indicates a p value < 0.001, ** < .01 * < .05.

| Supplemental Table 4: Unadjusted | | | | | | | | | | | | | | | | |
| --- | --- | --- | --- | --- | --- | --- | --- | --- | --- | --- | --- | --- | --- | --- | --- | --- |
|  | **Any mental disorder** | **Anxiety disorder** | **Depressive disorder** | **Personality disorder** | **Hyperkinetic disorder** | **Mental disorder NOS** | **Chronic alcohol abuse** | **Medication abuse** | **Drug abuse** | **Sleep disturbance** | **Affective psychosis** | **Psychosis** | **PTSD** | **Schizophrenia** | **Phobia** | **Suicide attempt** |
| **Intercept** | -0.57*** (0.01) | -2.32*** (0.03) | -1.61*** (0.02) | -3.62*** (0.05) | -2.79*** (0.04) | -3.12*** (0.04) | -3.14*** (0.05) | -3.50*** (0.07) | -2.35*** | -2.03*** (0.02) | -4.47*** (0.06) | -3.98*** | -4.10*** (0.07) | -3.71*** (0.06) | -3.39*** (0.05) | -3.73*** (0.07) |
| **Cognitive Abilities** | -0.17*** (0.00) | -0.20*** (0.01) | -0.14*** (0.00) | -0.22*** (0.01) | -0.31*** (0.01) | -0.22*** (0.01) | -0.27*** (0.01) | -0.39*** (0.02) | -0.35*** | -0.12*** (0.00) | -0.06*** (0.01) | -0.27*** | -0.25*** (0.02) | -0.24*** (0.01) | -0.18*** (0.01) | -0.35*** (0.02) |
| **AIC** | 263835.63 | 83417.57 | 163981.43 | 27457.60 | 38601.45 | 41036.42 | 33511.80 | 16527.36 | 48147.72 | 133401.38 | 26433.88 | 17319.53 | 16813.99 | 23869.32 | 38845.79 | 15846.65 |
| **BIC** | 263856.66 | 83438.60 | 164002.46 | 27478.63 | 38622.48 | 41057.45 | 33532.83 | 16548.39 | 48168.75 | 133422.41 | 26454.91 | 17340.56 | 16835.02 | 23890.35 | 38866.82 | 15867.68 |
| **Pseudo R2** | 0.02 | 0.02 | 0.01 | 0.01 | 0.03 | 0.02 | 0.02 | 0.04 | 0.04 | 0.01 | 0.00 | 0.02 | 0.02 | 0.02 | 0.01 | 0.03 |

| Supplemental Table 5: Adjusted for Parental Education and Income | | | | | | | | | | | | | | | | |
| --- | --- | --- | --- | --- | --- | --- | --- | --- | --- | --- | --- | --- | --- | --- | --- | --- |
|  | **Any mental disorder** | **Anxiety disorder** | **Depressive disorder** | **Personality disorder** | **Hyperkinetic disorder** | **Mental disorder NOS** | **Chronic alcohol abuse** | **Medication abuse** | **Drug abuse** | **Sleep disturbance** | **Affective psychosis** | **Psychosis** | **PTSD** | **Schizophrenia** | **Phobia** | **Suicide attempt** |
| **Intercept** | -0.55*** (0.02) | -2.21*** (0.04) | -1.48*** (0.02) | -3.61*** (0.07) | -2.87*** (0.06) | -3.10*** (0.06) | -2.98*** (0.09) | -3.21*** (0.09) | -2.19*** (0.05) | -2.07*** (0.03) | -4.61*** (0.08) | -4.13*** (0.09) | -4.12*** (0.10) | -4.10*** (0.08) | -3.33*** (0.06) | -3.56*** (0.10) |
| **Cognitive Abilities** | -0.17*** (0.00) | -0.19*** (0.01) | -0.13*** (0.00) | -0.21*** (0.01) | -0.31*** (0.01) | -0.23*** (0.01) | -0.26*** (0.01) | -0.36*** (0.02) | -0.35*** (0.01) | -0.12*** (0.00) | -0.08*** (0.01) | -0.28*** (0.02) | -0.25*** (0.02) | -0.28*** (0.01) | -0.17*** (0.01) | -0.33*** (0.02) |
| **Educational Attainment Mother** | -0.01** (0.00) | -0.04*** (0.01) | -0.02*** (0.00) | -0.02 (0.02) | 0.01 (0.01) | 0.00 (0.01) | 0.02 (0.01) | -0.08*** (0.02) | -0.03** (0.01) | -0.01* (0.01) | 0.02 (0.01) | 0.00 (0.02) | -0.01 (0.02) | 0.05*** (0.02) | 0.03** (0.01) | -0.05* (0.02) |
| **Educational Attainment Father** | -0.01** (0.00) | -0.01 (0.01) | -0.01** (0.00) | -0.00 (0.01) | -0.03* (0.01) | 0.03** (0.01) | -0.02 (0.01) | -0.05* (0.02) | -0.02** (0.01) | -0.01* (0.01) | 0.04** (0.01) | 0.05** (0.02) | -0.01 (0.02) | 0.09*** (0.01) | -0.03** (0.01) | -0.01 (0.02) |
| **Combined Income** | 0.00** (0.00) | 0.00 (0.00) | 0.00*** (0.00) | 0.00 (0.00) | 0.00*** (0.00) | 0.00*** (0.00) | -0.00* (0.00) | 0.00 (0.00) | 0.00 (0.00) | 0.00*** (0.00) | 0.00 (0.00) | 0.00 (0.00) | 0.00 (0.00) | 0.00*** (0.00) | 0.00*** (0.00) | 0.00 (0.00) |
| **AIC** | 263811.95 | 83387.38 | 163910.78 | 27454.71 | 38576.40 | 41019.13 | 33502.25 | 16505.10 | 48118.18 | 133337.56 | 26424.07 | 17315.62 | 16815.95 | 23794.97 | 38818.24 | 15843.89 |
| **BIC** | 263864.52 | 83439.96 | 163963.35 | 27507.29 | 38628.98 | 41071.70 | 33554.83 | 16557.68 | 48170.76 | 133390.13 | 26476.64 | 17368.19 | 16868.52 | 23847.55 | 38870.82 | 15896.46 |
| **Pseudo R2** | 0.02 | 0.02 | 0.01 | 0.02 | 0.03 | 0.02 | 0.02 | 0.04 | 0.04 | 0.01 | 0.00 | 0.02 | 0.02 | 0.02 | 0.01 | 0.03 |

| Supplemental Table 6: Adjusted for Parental Education, Income and Educational Attainment | | | | | | | | | | | | | | | | |
| --- | --- | --- | --- | --- | --- | --- | --- | --- | --- | --- | --- | --- | --- | --- | --- | --- |
|  | **Any mental disorder** | **Anxiety disorder** | **Depressive disorder** | **Personality disorder** | **Hyperkinetic disorder** | **Mental disorder NOS** | **Chronic alcohol abuse** | **Medication abuse** | **Drug abuse** | **Sleep disturbance** | **Affective psychosis** | **Psychosis** | **PTSD** | **Schizophrenia** | **Phobia** | **Suicide attempt** |
| **Intercept** | -0.27*** (0.02) | -1.94*** (0.04) | -1.24*** (0.03) | -3.15*** (0.07) | -2.40*** (0.06) | -2.81*** (0.06) | -2.59*** (0.07) | -2.58*** (0.10) | -1.48*** (0.05) | -1.87*** (0.03) | -4.31*** (0.08) | -3.83*** (0.10) | -3.81*** (0.10) | -3.73*** (0.08) | -3.06*** (0.06) | -3.22*** (0.10) |
| **Cognitive Abilities** | -0.13*** (0.00) | -0.15*** (0.01) | -0.10*** (0.00) | -0.16*** (0.01) | -0.24*** (0.01) | -0.19*** (0.01) | -0.20*** (0.01) | -0.26*** (0.02) | -0.21*** (0.01) | -0.09*** (0.00) | -0.03* (0.01) | -0.24*** (0.02) | -0.20*** (0.02) | -0.22*** (0.01) | -0.13*** (0.01) | -0.29*** (0.02) |
| **Educational Attainment Mother** | 0.01* (0.00) | -0.02* (0.01) | 0.01 (0.00) | 0.01 (0.02) | 0.04** (0.01) | 0.03* (0.01) | 0.01 (0.01) | 0.04* (0.02) | 0.00 (0.01) | 0.00 (0.01) | 0.04** (0.02) | 0.02 (0.02) | 0.02 (0.02) | 0.08*** (0.02) | 0.01 (0.01) | 0.03 (0.02) |
| **Educational Attainment Father** | 0.01*** (0.00) | 0.01 (0.01) | 0.01 (0.00) | 0.02 (0.01) | 0.01 (0.01) | 0.05*** (0.01) | 0.01 (0.01) | 0.00 (0.02) | 0.03** (0.01) | 0.00 (0.01) | 0.07*** (0.01) | 0.07*** (0.02) | 0.01 (0.02) | 0.12*** (0.01) | 0.01 (0.01) | 0.00 (0.02) |
| **Combined Income** | 0.00*** (0.00) | 0.00 (0.00) | -0.00*** (0.00) | 0.00 (0.00) | -0.00*** (0.00) | -0.00*** (0.00) | 0.00 (0.00) | 0.00 (0.00) | -0.00** (0.00) | 0.00*** (0.00) | 0.00 (0.00) | 0.00 (0.00) | 0.00* (0.00) | 0.00 (0.00) | 0.00*** (0.00) | -0.00 (0.00) |
| **Educational Attainment** | -0.14*** (0.00) | -0.14*** (0.01) | -0.12*** (0.00) | -0.20*** (0.01) | -0.25*** (0.01) | -0.15*** (0.01) | -0.21*** (0.01) | -0.35*** (0.02) | -0.42*** (0.01) | -0.10*** (0.00) | -0.17*** (0.01) | -0.15*** (0.02) | -0.16*** (0.02) | -0.20*** (0.01) | -0.14*** (0.01) | -0.17*** (0.02) |
| **AIC** | 261822.45 | 82908.52 | 163105.97 | 27231.15 | 38003.98 | 40791.28 | 33167.57 | 16140.29 | 46361.86 | 132901.08 | 26276.91 | 17321.42 | 16725.38 | 23586.47 | 38631.24 | 15744.35 |
| **BIC** | 261885.54 | 82971.61 | 163169.06 | 27294.24 | 38067.07 | 40854.36 | 33230.66 | 16203.38 | 46424.95 | 132964.17 | 26339.99 | 17294.51 | 16788.47 | 23649.56 | 38694.33 | 15807.44 |
| **Pseudo R2** | 0.03 | 0.02 | 0.02 | 0.02 | 0.05 | 0.02 | 0.03 | 0.06 | 0.08 | 0.01 | 0.01 | 0.02 | 0.02 | 0.03 | 0.02 | 0.04 |

| Supplemental Table 7: Within-Family Cognitive Abilities | | | | | | | | | | | | | | | | |
| --- | --- | --- | --- | --- | --- | --- | --- | --- | --- | --- | --- | --- | --- | --- | --- | --- |
|  | **Any mental disorder** | **Anxiety disorder** | **Depressive disorder** | **Personality disorder** | **Hyperkinetic disorder** | **Mental disorder NOS** | **Chronic alcohol abuse** | **Medication abuse** | **Drug abuse** | **Sleep disturbance** | **Affective psychosis** | **Psychosis** | **Schizophrenia** | **Phobia** | **PTSD** | **Suicide attempt** |
| **Intercept** | -0.34*** (0.03) | -2.13*** (0.06) | -1.37*** (0.02) | -3.04*** (0.11) | -2.26*** (0.09) | -2.68*** (0.09) | -2.52*** (0.09) | -2.56*** (0.15) | -1.17*** (0.08) | -1.84*** (0.04) | -3.95*** (0.11) | -3.54*** (0.14) | -3.16*** (0.11) | -3.03*** (0.09) | -3.78*** (0.14) | -3.42*** (0.15) |
| **Within Cognitive Abilities** | -0.06*** (0.01) | -0.08*** (0.02) | -0.03* (0.01) | -0.04 (0.04) | -0.14*** (0.03) | -0.09** (0.03) | -0.03 (0.04) | -0.10 (0.06) | -0.08** (0.03) | -0.06*** (0.01) | -0.08* (0.04) | -0.15** (0.05) | -0.14*** (0.04) | -0.05 (0.03) | -0.05 (0.05) | -0.10 (0.06) |
| **Educational Attainment** | -0.27*** (0.01) | -0.30*** (0.01) | -0.24*** (0.01) | -0.46*** (0.03) | -0.57*** (0.02) | -0.40*** (0.02) | -0.51*** (0.02) | -0.79*** (0.05) | -0.80*** (0.02) | -0.19*** (0.01) | -0.19*** (0.02) | -0.46*** (0.04) | -0.44*** (0.03) | -0.30*** (0.02) | -0.40*** (0.04) | -0.51*** (0.04) |
| **AIC** | 76496.43 | 23356.13 | 47016.73 | 6825.81 | 9594.64 | 11078.68 | 9190.51 | 4042.42 | 11705.51 | 39344.91 | 7937.14 | 4675.86 | 6812.88 | 11464.94 | 4620.90 | 4335.71 |
| **BIC** | 76524.46 | 23384.15 | 47044.75 | 6853.84 | 9622.67 | 11106.70 | 9218.54 | 4070.45 | 11733.53 | 39372.93 | 7965.16 | 4703.88 | 6840.91 | 11492.97 | 4648.93 | 4363.73 |

## Logistic regression while treating cognitive abilities as an ordinal variable

In addition to our main models presented in Figure 5 and Supplemental Table 4, 5, 6 and 7, we also modelled the same data while treating cognitive abilities as an ordinal variable. For this procedure we treated the 9 stanine levels as distinct factors using the following R code.

| # Convert cognitive abilities into a factor  dat$cog_ab <- factor(dat$cog_ab)  # Model using cognitive abilites as ordinal variable  glm(mental_disorder ~ cog_ab, family = binomial,data = dat) |
| --- |

The tables below contain the log-odds coefficients from each respective model, with the standard error in parenthesis. Cognitive ability stanine level 1 is treated as the reference category (intercept). In the plot below, CA stands for cognitive abilities.

| Supplemental Table 8: Unadjusted Model | | | | | | | | | | | | | | | | |
| --- | --- | --- | --- | --- | --- | --- | --- | --- | --- | --- | --- | --- | --- | --- | --- | --- |
|  | Any mental disorder | Anxiety disorder | Depressive disorder | Personality disorder | Hyperkinetic disorder | Mental disorder NOS | Chronic alcohol abuse | Medication abuse | Drug abuse | Sleep disturbance | Affective psychosis | Psychosis | PTSD | Schizophrenia | Phobia | Suicide attempt |
| **(Intercept)** | -0.715 | -2.607 | -1.801 | -3.714 | -3.297 | -3.116 | -3.349 | -4.155 | -2.962 | -2.294 | -4.514 | -4.166 | -4.601 | -3.692 | -3.648 | -3.973 |
|  | (0.028) | (0.053) | (0.038) | (0.087) | (0.072) | (0.066) | (0.074) | (0.108) | (0.062) | (0.046) | (0.129) | (0.109) | (0.134) | (0.086) | (0.085) | (0.099) |
| **CA2** | -0.226 | -0.103 | -0.061 | -0.401 | -0.111 | -0.397 | -0.429 | -0.151 | -0.143 | -0.060 | -0.032 | -0.346 | -0.026 | -0.525 | -0.121 | -0.581 |
|  | (0.034) | (0.063) | (0.046) | (0.111) | (0.087) | (0.083) | (0.093) | (0.131) | (0.075) | (0.055) | (0.153) | (0.136) | (0.160) | (0.112) | (0.102) | (0.130) |
| **CA3** | -0.345 | -0.266 | -0.210 | -0.594 | -0.356 | -0.730 | -0.573 | -0.527 | -0.376 | -0.056 | -0.254 | -0.642 | -0.268 | -0.848 | -0.246 | -0.835 |
|  | (0.032) | (0.059) | (0.043) | (0.102) | (0.081) | (0.078) | (0.086) | (0.125) | (0.070) | (0.051) | (0.145) | (0.128) | (0.151) | (0.105) | (0.095) | (0.120) |
| **CA4** | -0.512 | -0.509 | -0.344 | -0.785 | -0.717 | -0.941 | -0.911 | -0.783 | -0.703 | -0.153 | -0.134 | -0.887 | -0.376 | -0.937 | -0.443 | -1.104 |
|  | (0.030) | (0.057) | (0.041) | (0.098) | (0.080) | (0.075) | (0.083) | (0.121) | (0.068) | (0.049) | (0.137) | (0.123) | (0.145) | (0.098) | (0.092) | (0.115) |
| **CA5** | -0.716 | -0.687 | -0.510 | -1.013 | -1.045 | -1.165 | -1.166 | -1.166 | -1.060 | -0.338 | -0.225 | -1.168 | -0.723 | -1.276 | -0.686 | -1.465 |
|  | (0.030) | (0.057) | (0.041) | (0.098) | (0.081) | (0.075) | (0.084) | (0.123) | (0.069) | (0.049) | (0.136) | (0.124) | (0.147) | (0.100) | (0.092) | (0.117) |
| **CA6** | -0.897 | -0.873 | -0.665 | -1.357 | -1.459 | -1.378 | -1.395 | -1.826 | -1.476 | -0.453 | -0.322 | -1.400 | -0.996 | -1.555 | -0.836 | -1.839 |
|  | (0.031) | (0.059) | (0.042) | (0.104) | (0.086) | (0.078) | (0.088) | (0.139) | (0.074) | (0.050) | (0.138) | (0.130) | (0.153) | (0.106) | (0.094) | (0.128) |
| **CA7** | -1.057 | -1.123 | -0.822 | -1.434 | -1.753 | -1.525 | -1.732 | -2.322 | -1.985 | -0.607 | -0.362 | -1.673 | -1.300 | -1.641 | -0.970 | -2.273 |
|  | (0.032) | (0.063) | (0.044) | (0.112) | (0.098) | (0.086) | (0.100) | (0.174) | (0.089) | (0.052) | (0.143) | (0.147) | (0.169) | (0.116) | (0.101) | (0.157) |
| **CA8** | -1.168 | -1.286 | -0.893 | -1.654 | -1.983 | -1.657 | -1.921 | -2.806 | -2.649 | -0.673 | -0.517 | -2.100 | -1.641 | -1.843 | -1.178 | -2.616 |
|  | (0.035) | (0.072) | (0.048) | (0.134) | (0.121) | (0.100) | (0.121) | (0.248) | (0.130) | (0.056) | (0.155) | (0.192) | (0.206) | (0.140) | (0.115) | (0.211) |
| **CA9** | -1.321 | -1.524 | -0.939 | -1.659 | -2.035 | -1.774 | -2.580 | -2.993 | -2.574 | -0.849 | -0.389 | -1.924 | -2.061 | -1.869 | -1.480 | -2.856 |
|  | (0.042) | (0.095) | (0.056) | (0.170) | (0.160) | (0.133) | (0.206) | (0.370) | (0.170) | (0.068) | (0.173) | (0.235) | (0.308) | (0.182) | (0.155) | (0.317) |
| **AIC** | 263825.1 | 83424.5 | 163975.2 | 27461.4 | 38595.1 | 41025.0 | 33517.0 | 16516.2 | 48098.1 | 133378.3 | 26440.4 | 17329.9 | 16816.6 | 23862.1 | 38855.2 | 15855.4 |
| **BIC** | 263919.7 | 83519.1 | 164069.9 | 27556.0 | 38689.7 | 41119.7 | 33611.6 | 16610.8 | 48192.8 | 133472.9 | 26535.1 | 17424.6 | 16911.3 | 23956.7 | 38949.8 | 15950.1 |
| **Log.Lik.** | -131903.541 | -41703.248 | -81978.620 | -13721.699 | -19288.552 | -20503.524 | -16749.482 | -8249.081 | -24040.070 | -66680.144 | -13211.211 | -8655.961 | -8399.315 | -11922.030 | -19418.578 | -7918.711 |
| **RMSE** | 0.39 | 0.19 | 0.29 | 0.09 | 0.12 | 0.12 | 0.11 | 0.07 | 0.13 | 0.25 | 0.09 | 0.07 | 0.07 | 0.09 | 0.12 | 0.07 |

| Supplemental Figure 1: Unadjusted Model |
| --- |
| 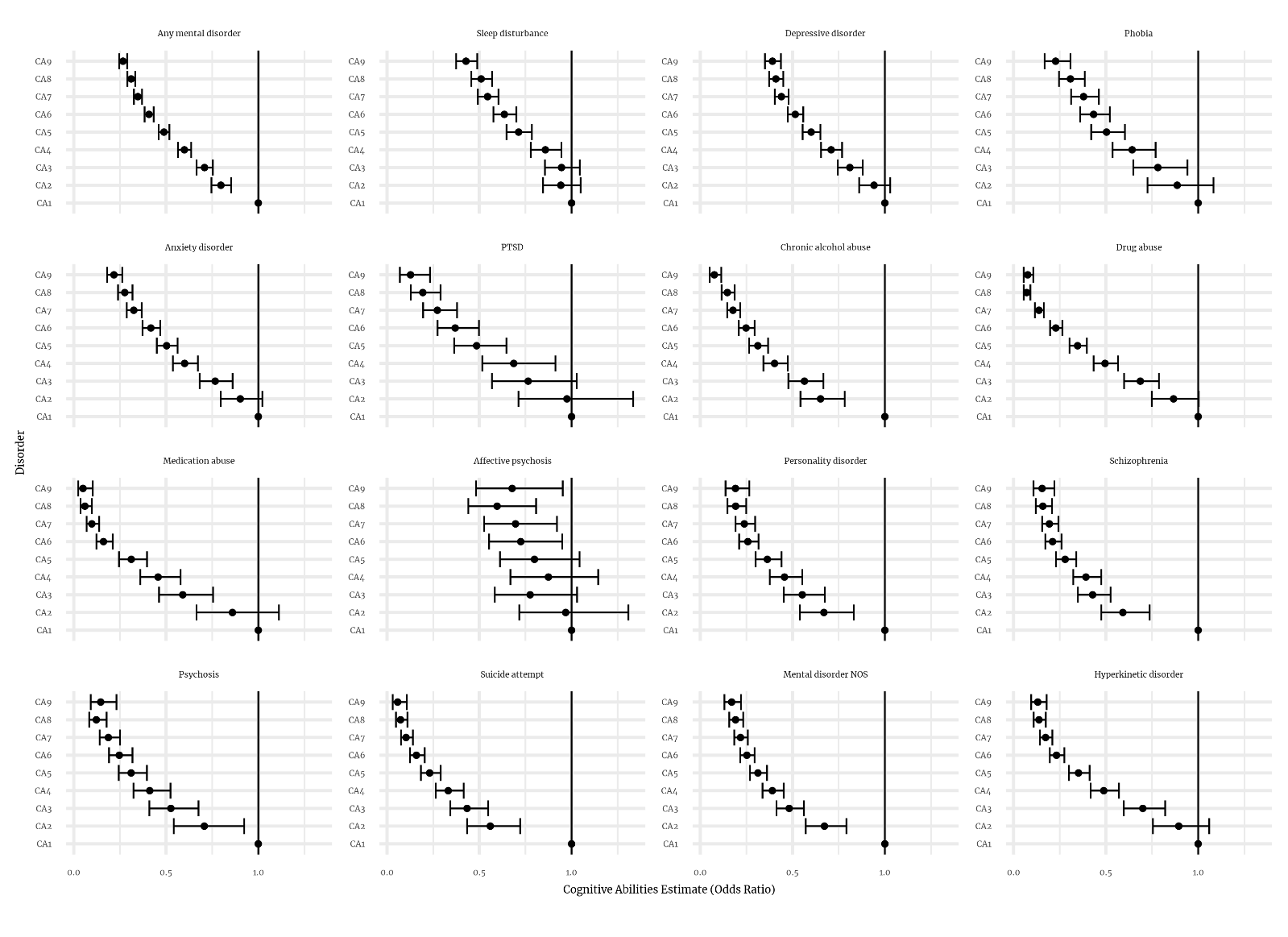 |
|  |

| Supplemental Table 9: Adjusted for Parental Education and Income | | | | | | | | | | | | | | | | |
| --- | --- | --- | --- | --- | --- | --- | --- | --- | --- | --- | --- | --- | --- | --- | --- | --- |
|  | Any mental disorder | Anxiety disorder | Depressive disorder | Personality disorder | Hyperkinetic disorder | Mental disorder NOS | Chronic alcohol abuse | Medication abuse | Drug abuse | Sleep disturbance | Affective psychosis | Psychosis | PTSD | Schizophrenia | Phobia | Suicide attempt |
| **(Intercept)** | -0.657 | -2.452 | -1.641 | -3.528 | -3.344 | -3.116 | -3.186 | -3.828 | -2.741 | -2.299 | -4.698 | -4.323 | -4.510 | -4.171 | -3.535 | -3.752 |
|  | (0.032) | (0.060) | (0.043) | (0.103) | (0.084) | (0.079) | (0.088) | (0.134) | (0.073) | (0.051) | (0.141) | (0.131) | (0.155) | (0.106) | (0.095) | (0.126) |
| **CA2** | -0.237 | -0.110 | -0.059 | -0.404 | -0.149 | -0.393 | -0.432 | -0.112 | -0.169 | -0.068 | -0.028 | -0.365 | -0.067 | -0.516 | -0.140 | -0.589 |
|  | (0.035) | (0.064) | (0.047) | (0.112) | (0.087) | (0.085) | (0.095) | (0.133) | (0.075) | (0.056) | (0.155) | (0.138) | (0.164) | (0.114) | (0.103) | (0.132) |
| **CA3** | -0.355 | -0.266 | -0.195 | -0.604 | -0.405 | -0.739 | -0.563 | -0.496 | -0.391 | -0.076 | -0.294 | -0.674 | -0.309 | -0.908 | -0.281 | -0.839 |
|  | (0.032) | (0.060) | (0.044) | (0.104) | (0.082) | (0.080) | (0.087) | (0.128) | (0.071) | (0.052) | (0.147) | (0.130) | (0.154) | (0.108) | (0.096) | (0.122) |
| **CA4** | -0.517 | -0.500 | -0.316 | -0.788 | -0.785 | -0.949 | -0.897 | -0.730 | -0.707 | -0.170 | -0.189 | -0.935 | -0.388 | -1.028 | -0.456 | -1.084 |
|  | (0.031) | (0.059) | (0.042) | (0.100) | (0.081) | (0.077) | (0.085) | (0.124) | (0.069) | (0.050) | (0.140) | (0.126) | (0.149) | (0.102) | (0.093) | (0.117) |
| **CA5** | -0.714 | -0.662 | -0.466 | -0.987 | -1.118 | -1.185 | -1.146 | -1.070 | -1.039 | -0.349 | -0.304 | -1.249 | -0.713 | -1.418 | -0.693 | -1.441 |
|  | (0.031) | (0.059) | (0.042) | (0.100) | (0.082) | (0.077) | (0.086) | (0.127) | (0.071) | (0.050) | (0.139) | (0.128) | (0.151) | (0.104) | (0.094) | (0.121) |
| **CA6** | -0.887 | -0.836 | -0.606 | -1.314 | -1.525 | -1.408 | -1.361 | -1.697 | -1.427 | -0.458 | -0.426 | -1.486 | -0.976 | -1.750 | -0.823 | -1.771 |
|  | (0.032) | (0.060) | (0.043) | (0.107) | (0.088) | (0.081) | (0.090) | (0.144) | (0.076) | (0.051) | (0.142) | (0.135) | (0.157) | (0.111) | (0.096) | (0.132) |
| **CA7** | -1.039 | -1.068 | -0.750 | -1.373 | -1.822 | -1.569 | -1.690 | -2.153 | -1.924 | -0.606 | -0.486 | -1.809 | -1.255 | -1.889 | -0.946 | -2.186 |
|  | (0.033) | (0.066) | (0.045) | (0.116) | (0.101) | (0.090) | (0.104) | (0.179) | (0.091) | (0.054) | (0.147) | (0.154) | (0.174) | (0.122) | (0.103) | (0.161) |
| **CA8** | -1.143 | -1.215 | -0.807 | -1.572 | -2.037 | -1.701 | -1.884 | -2.596 | -2.550 | -0.668 | -0.663 | -2.241 | -1.593 | -2.128 | -1.133 | -2.505 |
|  | (0.036) | (0.075) | (0.049) | (0.139) | (0.124) | (0.104) | (0.126) | (0.253) | (0.132) | (0.058) | (0.160) | (0.199) | (0.213) | (0.146) | (0.118) | (0.216) |
| **CA9** | -1.286 | -1.420 | -0.838 | -1.565 | -2.130 | -1.842 | -2.501 | -2.732 | -2.445 | -0.833 | -0.583 | -2.065 | -1.965 | -2.223 | -1.406 | -2.716 |
|  | (0.044) | (0.098) | (0.059) | (0.177) | (0.166) | (0.138) | (0.210) | (0.374) | (0.172) | (0.070) | (0.180) | (0.242) | (0.314) | (0.189) | (0.158) | (0.323) |
| **Education Length Mother** | -0.014 | -0.047 | -0.029 | -0.036 | 0.020 | 0.013 | -0.010 | -0.076 | -0.042 | -0.015 | 0.034 | 0.010 | -0.031 | 0.070 | -0.027 | -0.041 |
|  | (0.004) | (0.009) | (0.005) | (0.017) | (0.013) | (0.013) | (0.015) | (0.024) | (0.012) | (0.006) | (0.016) | (0.022) | (0.023) | (0.017) | (0.014) | (0.024) |
| **Education Length Father** | -0.013 | -0.009 | -0.015 | -0.025 | -0.033 | 0.024 | -0.024 | -0.051 | -0.035 | -0.014 | 0.041 | 0.043 | -0.032 | 0.083 | -0.037 | -0.031 |
|  | (0.004) | (0.008) | (0.005) | (0.016) | (0.013) | (0.012) | (0.014) | (0.022) | (0.011) | (0.006) | (0.015) | (0.020) | (0.021) | (0.016) | (0.012) | (0.022) |
| **Combined Income** | 0.001 | 0.000 | -0.001 | 0.000 | 0.003 | -0.002 | -0.001 | 0.000 | 0.001 | 0.002 | 0.000 | 0.001 | 0.002 | 0.001 | 0.002 | 0.000 |
|  | (0.000) | (0.000) | (0.000) | (0.001) | (0.001) | (0.001) | (0.001) | (0.001) | (0.001) | (0.000) | (0.001) | (0.001) | (0.001) | (0.001) | (0.001) | (0.001) |
| **AIC** | 260044.0 | 82076.5 | 161562.5 | 26948.0 | 37970.2 | 40402.2 | 33099.1 | 16269.4 | 47280.9 | 131396.5 | 26158.8 | 16986.3 | 16359.5 | 23313.9 | 38394.7 | 15604.3 |
| **BIC** | 260170.1 | 82202.5 | 161688.6 | 27074.0 | 38096.2 | 40528.2 | 33225.2 | 16395.4 | 47406.9 | 131522.6 | 26284.9 | 17112.4 | 16485.5 | 23440.0 | 38520.7 | 15730.3 |
| **Log.Lik.** | -130010.013 | -41026.253 | -80769.274 | -13461.985 | -18973.081 | -20189.089 | -16537.568 | -8122.701 | -23628.436 | -65686.269 | -13067.423 | -8481.172 | -8167.744 | -11644.963 | -19185.342 | -7790.136 |
| **RMSE** | 0.39 | 0.19 | 0.29 | 0.09 | 0.12 | 0.12 | 0.11 | 0.07 | 0.13 | 0.25 | 0.09 | 0.07 | 0.07 | 0.09 | 0.12 | 0.07 |

| Supplemental Figure 2: Ordinal Model Adjusted for Parental Education and Income |
| --- |
| 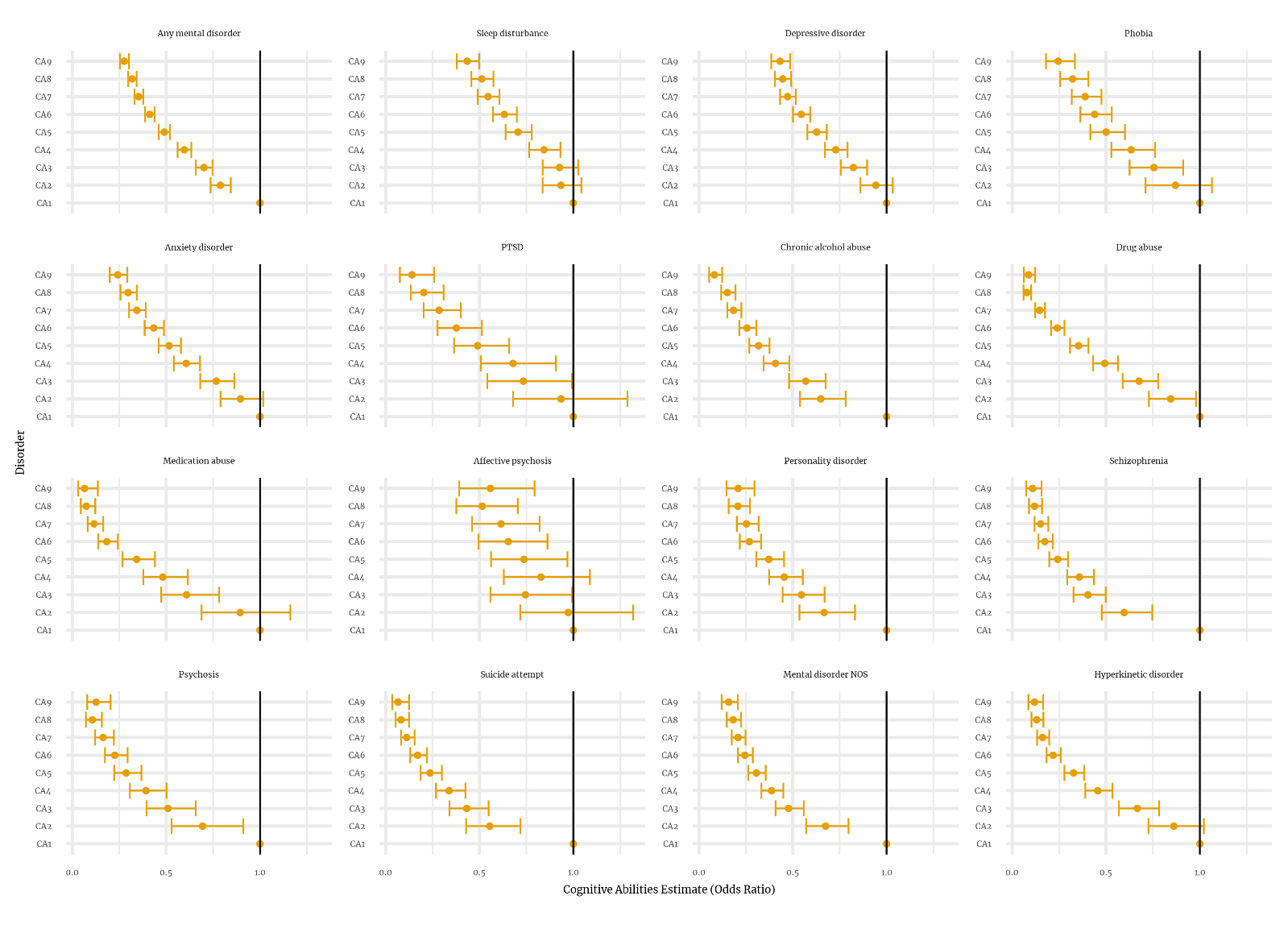 |
|  |

| Supplemental Table 10: Adjusted for Parental Education, Income and Educational Attainment | | | | | | | | | | | | | | | | |
| --- | --- | --- | --- | --- | --- | --- | --- | --- | --- | --- | --- | --- | --- | --- | --- | --- |
|  | Any mental disorder | Anxiety disorder | Depressive disorder | Personality disorder | Hyperkinetic disorder | Mental disorder NOS | Chronic alcohol abuse | Medication abuse | Drug abuse | Sleep disturbance | Affective psychosis | Psychosis | PTSD | Schizophrenia | Phobia | Suicide attempt |
| **(Intercept)** | -0.243 | -2.028 | -1.256 | -2.847 | -2.396 | -2.558 | -2.595 | -2.775 | -1.467 | -2.009 | -4.244 | -3.782 | -3.830 | -3.546 | -3.122 | -3.111 |
|  | (0.036) | (0.069) | (0.049) | (0.123) | (0.096) | (0.093) | (0.105) | (0.158) | (0.086) | (0.058) | (0.160) | (0.160) | (0.172) | (0.128) | (0.110) | (0.151) |
| **CA2** | -0.157 | -0.037 | -0.020 | -0.264 | -0.116 | -0.280 | -0.231 | 0.095 | -0.016 | -0.022 | 0.026 | -0.168 | -0.186 | -0.315 | -0.044 | -0.396 |
|  | (0.039) | (0.072) | (0.052) | (0.130) | (0.097) | (0.097) | (0.109) | (0.152) | (0.085) | (0.063) | (0.174) | (0.166) | (0.182) | (0.135) | (0.116) | (0.153) |
| **CA3** | -0.175 | -0.112 | -0.072 | -0.304 | -0.193 | -0.481 | -0.221 | -0.123 | -0.006 | 0.032 | -0.166 | -0.273 | -0.177 | -0.515 | -0.080 | -0.478 |
|  | (0.036) | (0.068) | (0.049) | (0.120) | (0.091) | (0.091) | (0.101) | (0.146) | (0.080) | (0.058) | (0.165) | (0.155) | (0.168) | (0.127) | (0.109) | (0.142) |
| **CA4** | -0.236 | -0.241 | -0.104 | -0.311 | -0.394 | -0.553 | -0.426 | -0.147 | -0.098 | 0.010 | 0.086 | -0.389 | -0.137 | -0.468 | -0.173 | -0.565 |
|  | (0.035) | (0.066) | (0.047) | (0.116) | (0.089) | (0.088) | (0.099) | (0.143) | (0.079) | (0.057) | (0.157) | (0.151) | (0.162) | (0.121) | (0.106) | (0.138) |
| **CA5** | -0.319 | -0.292 | -0.157 | -0.325 | -0.500 | -0.634 | -0.493 | -0.239 | -0.161 | -0.094 | 0.074 | -0.536 | -0.300 | -0.670 | -0.278 | -0.750 |
|  | (0.035) | (0.066) | (0.048) | (0.117) | (0.091) | (0.089) | (0.100) | (0.146) | (0.080) | (0.057) | (0.157) | (0.154) | (0.165) | (0.123) | (0.107) | (0.142) |
| **CA6** | -0.387 | -0.363 | -0.204 | -0.450 | -0.703 | -0.722 | -0.558 | -0.587 | -0.291 | -0.130 | 0.051 | -0.573 | -0.420 | -0.811 | -0.300 | -0.878 |
|  | (0.036) | (0.069) | (0.049) | (0.124) | (0.098) | (0.094) | (0.106) | (0.161) | (0.086) | (0.058) | (0.160) | (0.161) | (0.173) | (0.131) | (0.111) | (0.153) |
| **CA7** | -0.444 | -0.487 | -0.260 | -0.357 | -0.807 | -0.718 | -0.711 | -0.830 | -0.522 | -0.218 | 0.098 | -0.751 | -0.570 | -0.790 | -0.317 | -1.153 |
|  | (0.038) | (0.074) | (0.051) | (0.134) | (0.110) | (0.102) | (0.118) | (0.194) | (0.100) | (0.061) | (0.166) | (0.179) | (0.191) | (0.142) | (0.118) | (0.182) |
| **CA8** | -0.476 | -0.575 | -0.255 | -0.422 | -0.889 | -0.744 | -0.817 | -1.086 | -0.970 | -0.228 | -0.017 | -1.141 | -0.772 | -0.905 | -0.434 | -1.315 |
|  | (0.041) | (0.083) | (0.055) | (0.155) | (0.133) | (0.117) | (0.140) | (0.265) | (0.140) | (0.065) | (0.179) | (0.227) | (0.228) | (0.165) | (0.132) | (0.232) |
| **CA9** | -0.547 | -0.695 | -0.222 | -0.227 | -0.750 | -0.790 | -1.294 | -1.117 | -0.629 | -0.340 | 0.154 | -0.762 | -1.026 | -0.840 | -0.598 | -1.471 |
|  | (0.048) | (0.105) | (0.064) | (0.191) | (0.174) | (0.150) | (0.222) | (0.406) | (0.185) | (0.077) | (0.199) | (0.266) | (0.325) | (0.206) | (0.169) | (0.349) |
| **Education Length Mother** | 0.020 | -0.013 | 0.002 | 0.017 | 0.077 | 0.063 | 0.038 | -0.012 | 0.034 | 0.008 | 0.069 | 0.063 | 0.020 | 0.127 | 0.009 | 0.003 |
|  | (0.004) | (0.009) | (0.006) | (0.018) | (0.014) | (0.013) | (0.015) | (0.025) | (0.013) | (0.006) | (0.017) | (0.023) | (0.023) | (0.018) | (0.014) | (0.025) |
| **Education Length Father** | 0.027 | 0.035 | 0.020 | 0.045 | 0.042 | 0.076 | 0.043 | 0.047 | 0.059 | 0.013 | 0.089 | 0.113 | 0.020 | 0.149 | 0.011 | 0.046 |
|  | (0.004) | (0.008) | (0.005) | (0.016) | (0.013) | (0.012) | (0.014) | (0.022) | (0.012) | (0.006) | (0.016) | (0.021) | (0.022) | (0.017) | (0.013) | (0.023) |
| **Combined Income** | 0.001 | 0.001 | 0.000 | 0.001 | 0.005 | -0.002 | 0.000 | 0.002 | 0.002 | 0.003 | 0.000 | 0.001 | 0.003 | 0.002 | 0.003 | 0.000 |
|  | (0.000) | (0.000) | (0.000) | (0.001) | (0.001) | (0.001) | (0.001) | (0.001) | (0.001) | (0.000) | (0.001) | (0.001) | (0.001) | (0.001) | (0.001) | (0.001) |
| **Educational Attainment** | -0.259 | -0.264 | -0.225 | -0.460 | -0.531 | -0.369 | -0.422 | -0.663 | -0.747 | -0.174 | -0.276 | -0.433 | -0.367 | -0.460 | -0.280 | -0.446 |
|  | (0.004) | (0.008) | (0.005) | (0.017) | (0.014) | (0.013) | (0.015) | (0.025) | (0.013) | (0.006) | (0.016) | (0.023) | (0.023) | (0.018) | (0.013) | (0.024) |
| **AIC** | 243338.5 | 76712.3 | 151841.7 | 24144.6 | 33709.3 | 36677.7 | 30203.2 | 14417.1 | 40651.0 | 124499.3 | 24521.9 | 15165.2 | 14997.1 | 20929.6 | 35801.5 | 14046.3 |
| **BIC** | 243474.5 | 76848.3 | 151977.7 | 24280.6 | 33845.3 | 36813.7 | 30339.2 | 14553.1 | 40787.1 | 124635.3 | 24658.0 | 15301.2 | 15133.1 | 21065.6 | 35937.5 | 14182.3 |
| **Log.Lik.** | -121656.227 | -38343.171 | -75907.835 | -12059.306 | -16841.642 | -18325.845 | -15088.589 | -7195.538 | -20312.524 | -62236.635 | -12247.972 | -7569.615 | -7485.528 | -10451.800 | -17887.760 | -7010.145 |
| **RMSE** | 0.39 | 0.18 | 0.28 | 0.09 | 0.11 | 0.12 | 0.10 | 0.07 | 0.13 | 0.25 | 0.09 | 0.07 | 0.07 | 0.08 | 0.11 | 0.07 |

| Supplemental Figure 3: Ordinal Model Adjusted for Parental Education, Income and Educational Attainment |
| --- |
| 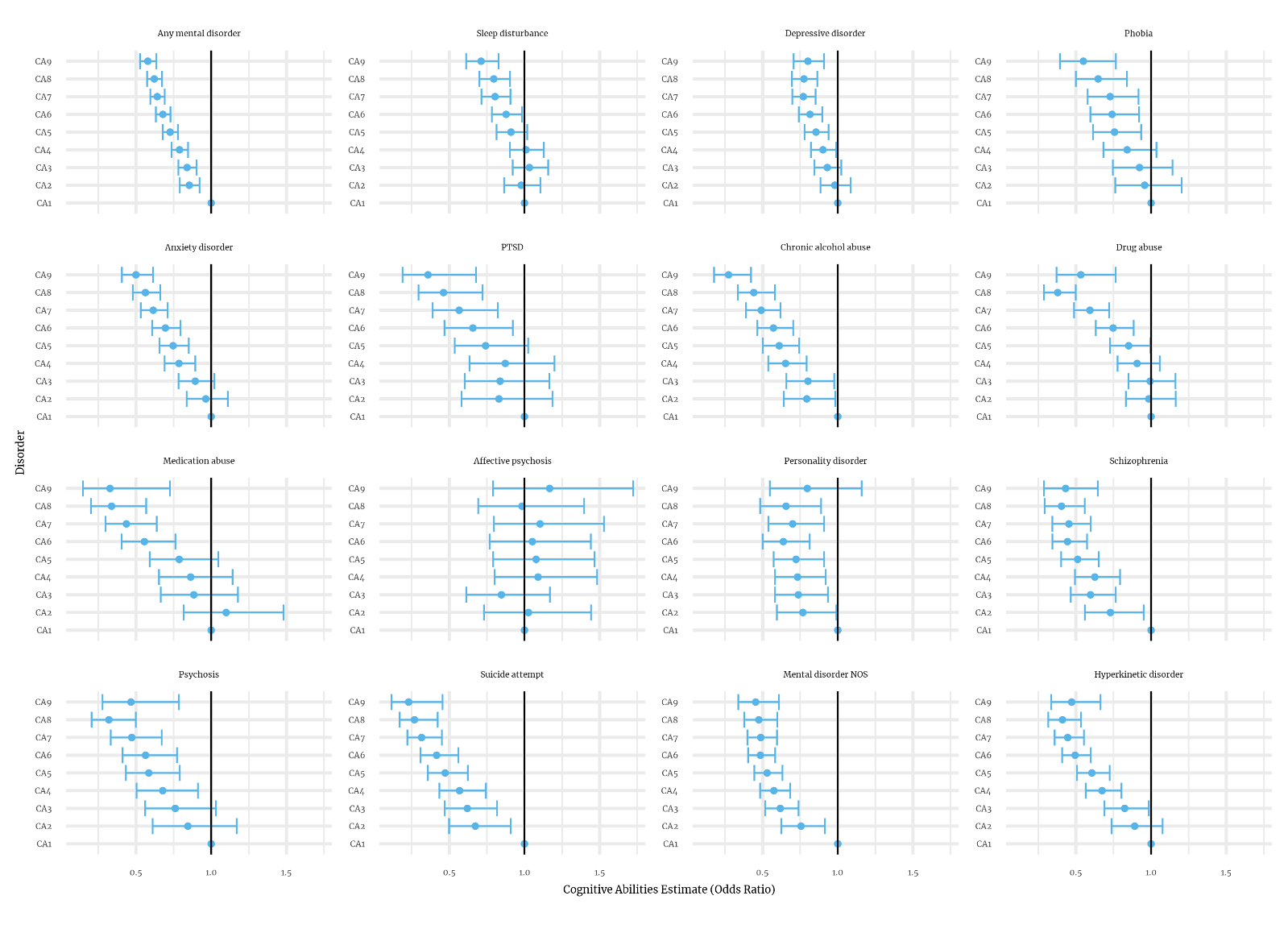 |
|  |

| Supplemental Table 11: ICPC-2 Codes The table below was copied from the ​ICPC-2e-v.7.0, taken from the official website <https://icpc2.icpc-3.info/>. | | | |
| --- | --- | --- | --- |
| **code** | **name** | **criteria** | **icd10** |
| P70 | Dementia | a syndrome due to a disease of the brain, usually of a chronic and/or progressive nature, with clinically significant disturbance of multiple higher cortical functions (memory, thinking, orientation, comprehension), together with intact consciousness | F00.0; F00.1; F00.2; F00.9; F01.0; F01.1; F01.2; F01.3; F01.8; F01.9; F02.0; F02.1; F02.2; F02.3; F02.4; F02.8; F03; G30.0; G30.1; G30.8; G30.9 |
| P71 | Organic psychosis other | organic psychiatric disorders as a diagnosis require psychological syndromes, patterns or behaviour due to organic disease | F04; F05.0; F05.1; F05.8; F05.9; F06.0; F06.1; F06.2; F06.3; F06.4; F06.5; F06.6; F06.7; F06.8; F06.9; F07.0; F07.1; F07.2; F07.8; F07.9; F09 |
| P72 | Schizophrenia | fundamental and characteristic distortions of thinking, perception and affect that are inappropriate or blunted (eg. thought-echo, -insertion, -withdrawal, delusional perceptions, hallucinatory voices, delusions of control), together with a clear consciousness and unaffected intellectual capacity. | F20.0; F20.1; F20.2; F20.3; F20.4; F20.5; F20.6; F20.8; F20.9; F21; F22.0; F22.8; F22.9; F24; F25.0; F25.1; F25.2; F25.8; F25.9; F28 |
| P73 | Affective psychosis | a fundamental disturbance in affect and mood (with/without associated anxiety). In manic disorder mood, energy and activity are simultaneously elevated. In bipolar disease, at least two periods of disturbed mood, shifting from elevated to lowered are observed | F30.0; F30.1; F30.2; F30.8; F30.9; F31.0; F31.1; F31.2; F31.3; F31.4; F31.5; F31.6; F31.7; F31.8; F31.9; F34.0 |
| P74 | Anxiety disorder/anxiety state | clinically significant anxiety that is not restricted to any particular environmental situation. It manifests as a panic disorder (recurrent attacks of severe anxiety not restricted to any particular situation, with or without physical symptoms) or as a disorder in which generalized and persistent anxiety, not related to any particular situation, occurs with variable physical symptoms | F41.0; F41.1; F41.3; F41.8; F41.9 |
| P75 | Somatization disorder | somatization disorder is characterized by a preoccupation with and repeated presentations of physical symptoms and complaints together with persistent requests for medical investigations in spite of repeated negative findings and reassurances by doctors. For this diagnosis, the presentation of multiple, recurrent and frequently changing physical symptoms presented to the family physician over a period of at least one year is required. Hypochondriacal disorder requires a persistent preoccupation with either the physical appearance or with the possibility of having a serious disease, together with persistent somatic complaints over a period of at least one year, in spite of repeated negative findings and reassurances by doctors | F44.0; F44.1; F44.2; F44.3; F44.4; F44.5; F44.6; F44.7; F44.8; F44.9; F45.0; F45.1; F45.2; F45.3; F45.4; F45.9 |
| P76 | Depressive disorder | fundamental disturbance in affect and mood towards depression. Mood, energy and activity are simultaneously lowered, together with an impaired capacity for enjoyment, interest and concentration. Sleep and appetite are usually disturbed and self-esteem and confidence are decreased | F32.0; F32.1; F32.2; F32.3; F32.8; F32.9; F33.0; F33.1; F33.2; F33.3; F33.4; F33.8; F33.9; F34.1; F34.8; F34.9; F38.0; F38.1; F38.8; F39; F41.2; F53.0 |
| P77 | Suicide/suicide attempt |  | Z91.5 |
| P78 | Neuraesthenia/surmenage | increased fatigueability with unpleasant associations, difficulties in concentration and a persistent decrease in performance and coping efficiency; the feeling of physical weakness and exhaustion after mental effort or after a minimal physical effort is often accompanied by muscular pain and an inability to relax | F48.0 |
| P79 | Phobia/compulsive disorder | phobic anxiety disorder requires outspoken anxiety, evoked only in well defined situations that are not generally considered dangerous: the patient tries to avoid these situations, or endures them with dread. Obsessive compulsive disorder requires distressing and recurrent obsessional thoughts/acts recognized by the patient as his/her own; compulsive stereotyped behaviours are repeated again and again, intended to prevent some objective unlikely event and recognized by the patient as pointless and ineffective | F40.0; F40.1; F40.2; F40.8; F40.9; F42.0; F42.1; F42.2; F42.8; F42.9 |
| P80 | Personality disorder | persistent and clinically important conditions and behaviour patterns in an individual's lifestyle and mode of relating to him/herself and others, reflecting significant/extreme deviations from the way an average individual in a given culture perceives, feels and behaves. This pattern is deeply ingrained and longlasting | F60.0; F60.1; F60.2; F60.3; F60.4; F60.5; F60.6; F60.7; F60.8; F60.9; F61; F62.0; F62.1; F62.8; F62.9; F63.0; F63.1; F63.2; F63.8; F63.9; F68.0; F68.1; F68.8; F69 |
| P81 | Hyperkinetic disorder | early onset of a lack of persistence in activities requiring cognitive involvement, with a tendency to move from one activity to another without completing any one, with disorganised and ill-regulated behaviour, and excessive activity | F90.0; F90.1; F90.8; F90.9 |
| P82 | Post-traumatic stress disorder | a stressful event followed by a major state of distress and disturbance, with a delayed or protracted reaction, flashbacks, nightmares, emotional blunting and anhedonia interfering with social functioning and performance, and including depressed mood, anxiety, worry and feeling unable to cope, persistent over time | F43.1 |
| P85 | Mental retardation | arrested/incomplete development of the mind with impairment of skills during the developmental period, and a low overall level of intelligence, with/without impairment of behaviour. | F70.0; F70.1; F70.8; F70.9; F71.0; F71.1; F71.8; F71.9; F72.0; F72.1; F72.8; F72.9; F73.0; F73.1; F73.8; F73.9; F78.0; F78.1; F78.8; F78.9; F79.0; F79.1; F79.8; F79.9 |
| P86 | Anorexia nervosa/bulimia | Anorexia nervosa: deliberate weight loss induced and sustained by the patient, associated with an intensive and overvalued dread of fatness and flabbiness of body contours. Bulimia: repeated bouts of overeating and an excessive preoccupation with body weight, leading to a pattern of overeating followed by induced vomiting or use of purgatives | F50.0; F50.1; F50.2; F50.3; F50.4 |
| P98 | Psychosis NOS/other |  | F23.0; F23.1; F23.2; F23.3; F23.8; F23.9; F29; F53.1 |
| P99 | Psychological disorders other |  | F48.1; F48.8; F48.9; F53.8; F53.9; F54; F59; F84.0; F84.1; F84.2; F84.3; F84.4; F84.5; F84.8; F84.9; F88; F89; F99 |
